# Site-specific genetic and functional signatures of aortic endothelial cells at aneurysm predilection sites in healthy and AngII ApoE^−/−^ mice

**DOI:** 10.1007/s10456-024-09933-9

**Published:** 2024-07-04

**Authors:** Alexander Brückner, Adrian Brandtner, Sarah Rieck, Michaela Matthey, Caroline Geisen, Benedikt Fels, Marta Stei, Kristina Kusche-Vihrog, Bernd K. Fleischmann, Daniela Wenzel

**Affiliations:** 1 Life&Brain Center, Medical Faculty, Institute of Physiology I, University of Bonn, Bonn, Germany; 2 Institute of Physiology, University of Lübeck, Lübeck, Germany; 3 DZHK (German Research Centre for Cardiovascular Research), Partner SiteHamburg/Luebeck/Kiel, Luebeck, Germany; 4 Heart Center Bonn, Clinic for Internal Medicine II, University Hospital Bonn, Bonn, Germany; 5 Department of Systems Physiology, Medical Faculty, Institute of Physiology, Ruhr University of Bochum, Universitätsstr. 150, 44801 Bochum, Germany

**Keywords:** Aortic aneurysm, Endothelial cells, Heterogeneity, Site-specificity, RNA-seq

## Abstract

Aortic aneurysm is characterized by a pathological dilation at specific predilection sites of the vessel and potentially results in life-threatening vascular rupture. Herein, we established a modified “Häutchen method” for the local isolation of endothelial cells (ECs) from mouse aorta to analyze their spatial heterogeneity and potential role in site-specific disease development. When we compared ECs from aneurysm predilection sites of healthy mice with adjacent control segments we found regulation of genes related to extracellular matrix remodeling, angiogenesis and inflammation, all pathways playing a critical role in aneurysm development. We also detected enhanced cortical stiffness of the endothelium at these sites. Gene expression of ECs from aneurysms of the AngII ApoE^−/−^ model when compared to sham animals mimicked expression patterns from predilection sites of healthy animals. Thus, this work highlights a striking genetic and functional regional heterogeneity in aortic ECs of healthy mice, which defines the location of aortic aneurysm formation in disease.

**Supplementary Information:**

The online version contains supplementary material available at 10.1007/s10456-024-09933-9.

## Introduction

Aortic aneurysms are defined as focal dilations of the aorta. Extensive aortic enlargement carries the risk of vascular rupture, which has a high mortality rate. Aortic aneurysms preferentially develop at specific predilection sites in humans and also in mouse models. Abdominal aortic aneurysms (AAA) develop below the diaphragm, whereas thoracic aortic aneurysms (TAA) are most commonly found in the aortic root or ascending aortic arch [1]. AAA have a high prevalence in industrialized countries and are associated with other cardiovascular diseases [2], whereas TAA are less frequent and often occur in the context of genetic syndromes such as Marfan- or Loeys-Dietz Syndrome [3]. AAA and TAA are considered to be distinct pathophysiologic entities, because the affected aortic segments are of different embryonic origin, the structure of the vascular wall differs, there is disparity in protease and chemokine signaling pathways and also shear stress profiles are distinct [4]. A key finding of AA formation is structural degeneration of the aortic wall, therefore many studies in the past focused on changes of the smooth muscle layer and the extracellular matrix [5, 6]. Recent evidence in humans and also animal models, however, suggests that AAA is also strongly associated with endothelial dysfunction [7, 8]. Moreover, ECs have been reported to display a pronounced heterogeneity in different organs and there is even site-specific heterogeneity along the vascular tree within the same organ [9, 10]. Based on these findings, we hypothesized that there could be regional heterogeneity of ECs in the healthy aorta, which predisposes specific sites to AA formation. However, this question is difficult to address given the low number of ECs in the aortic wall and the need for site-specific isolation. We have therefore established a modified “Häutchen method” that enabled us to isolate highly enriched ECs from specific segments of mouse aorta. Using in depth bulk RNA-seq analysis of aortic ECs we demonstrate prominent transcriptomic heterogeneity between the different sites along the healthy aortic tree. At the AA predilection sites of healthy mice we found regulation of genes related to extracellular matix (ECM) remodeling, angiogenesis and inflammation. Interestingly, this expression pattern reflected genetic and structural changes at the sites of AA development in the AngII ApoE^−/−^ aneurysm model. Our data suggest that EC heterogeneity and dysfunction point towards the site-specificity of aneurysm formation.

## Methods

### Isolation of ECs from different aortic localizations using the modified “Häutchen method”

For the site-specific isolation of aortic ECs we opened the thoracic and abdominal cavity of male healthy C57BL/6 mice (10–13 weeks) or AngII ApoE^−/−^ or sham ApoE^−/−^ mice (see below). Then, the aorta was dissected free of connective tissue and perfused with heparin (250 i.E./ml). After that the whole aorta was isolated and cut into 4 segments representing the ascending and descending part of the aortic arch as well as the thoracic and abdominal part of the straight aorta. Intimal ECs and medial/adventitial cells were isolated using a modified “Häutchen method” [11]. “Häutchen methods” have been originally established to isolate endothelial monolayers of vessels after fixation for en face microscopy and Hirsch et al. developed a complex procedure to expose both sides of the endothelium of fixated vessels for autoradiography [12]. Our modified “Häutchen method” specifically applies cold to make surface cell layers adhere to glass and enable their isolation. To this aim, the ring-like aortic segments were cut open and positioned with the endothelial site down on top of a 12 mm glass cover slip. Then, another 12 mm glass cover slip that was pre-cooled in isopentane on dry ice was placed on the adventitial site of the aortic segment. Immediately, a pre-cooled copper rod (6 mm diameter) was pressed on top of this sandwich for a period of 10 s causing the transfer of the superficial cell layers to the glasses via mechanical force. Thereby, we isolated the endothelial and medial/adventitial cell layers adhering to one of the 2 separate cover slips, respectively. The remaining aortic tissue was discarded. The coverslips were rinsed with RLT buffer from the RNeasy Plus micro kit (Qiagen, Hilden, Germany) for RNA isolation of ECs or medial/adventitial cells. The entire procedure from sacrificing the animal to the lysing of the isolated cells did take maximally 25 min. Lysates were stored at −80 °C until use. Alternatively, cells adhering to the glasses were stained and counted.

### AngII ApoE^−/−^ mouse model for aneurysm formation

Male ApoE^−/−^ mice (10–18 weeks) were obtained from the Jackson laboratory (B6.129P2-Apoe^tm1Unc^/J), fed a standard laboratory chow and randomly assigned to the AngII or control group. Alzet osmotic mini pumps (Model 1004) were implanted to deliver 1000 ng/kg/min of Angiotensin II (Sigma-Aldrich) for a period of 14 or 28 days. Three days before implantation a western diet (1.25% Cholesterol, ssniff) was started. Disease progression was monitored using a Vevo 3100 ultrasound machine (Visual Sonics, Toronto, Canada). As controls, ApoE^−/−^ animals subjected to sham surgeries and western diet were used. All procedures were approved by the local government authorities (LANUV, NRW, Germany). In accordance with earlier studies from other groups we have used males mice, as akin to human males, they are more susceptible to aneurysm formation [13]. In addition, when focusing on fundamental pathogenic processes the standardized use of males helps to overcome biases, to enhance reproducibility and comparability across aneurysm studies [14].

### RNA isolation

RNA was isolated using the RNeasy Plus micro kit according to manufacturer’s instruction (Qiagen, Hilden, Germany). To assess RNA quality the RNA integrity number (RIN) was determined by a 2100 Bioanalyzer (Agilent Technologies, Santa Clara, CA, USA). Only samples with a RIN above 5.0 were processed further. There were no differences in the mean RIN values of the groups compared.

### qPCR analysis

QPCR analysis was performed as reported before [15, 16]. For reverse transcription the SuperScript VILO cDNA synthesis kit (LifeTechnologies) was used. Expression of murine Cd31 (QT01052044, Qiagen), VwF (QT00116795, Qiagen), Cdh5 (QT00110467, Qiagen) and 18SrRNA (QT01036875, Quiagen) was determined by QuantiTect Primer Assays (Qiagen) together with the QuantiNova DNA polymerase (QuantiNova SYBR Green PCR kit, Qiagen).

### Digital PCR (dPCR)

DPCR has been chosen because it requires very low amounts of template. Therefore, analysis could be performed in the same samples that had been applied for RNA-seq experiments. Nevertheless, in some of the samples there was not enough RNA left for dPCR, these had to be excluded. For dPCR, RNA from the isolated EC samples was first transcribed into cDNA using the SuperScript VILO cDNA synthesis kit (LifeTechnologies). This cDNA was then applied for digital PCR in a plate (QIAcuity Nanoplate 8.5 k 24-well) using a reaction mixture containing FAM-labeled Taqman probes for the target genes. Following assays were used: Aqp1 (Mm00431834_m1, ThermoFisher), Cdh11 (Mm00515466_m1, ThermoFisher), C7 (Mm01297045_m1, ThermoFisher) and Grem2 (Mm00501909_m1, ThermoFisher), Hand2 (Mm00439247_m1, ThermoFisher), Efemp1 (Mm01434321_m1, ThermoFisher), Cd55 (Mm00438377_m1, ThermoFisher), Ptn (Mm01132688_m1, ThermoFisher), Hoxc10 (Mm01305933_m1, ThermoFisher), Cfd (Mm01143935_g1, ThermoFisher) and Cidec (Mm00617672_m1, ThermoFisher). The Hprt gene was used as a housekeeper and was detected using a VIC labeled Taqman probe (Mm03024075_m1, ThermoFisher). Partitioning and imaging (exposure time: 500 ms, gain: 6) were performed automatically in the QIAcuity One instrument for endpoint PCR after 40 cycles.

The copy number of the target genes was normalized to the housekeeper Hprt.

### Histology

Aortic segments from were fixated with 4% PFA for 30 min and frozen in TissueTek. Then, 10 µm thick cryosection were generated with a cryotome (CM3050S, Leica, Wetzlar, Germany). Hematoxylin and eosin stainings were performed on aortic segments from male AngII ApoE^−/−^ or sham mice. Sections were then embedded with Entellan (Sigma-Aldrich) and pictures were taken with a Keyence BZ-X800 microscope (Keyence, Osaka, Japan) at 20 × magnification.

### Immunohistochemistry

Immunohistochemistry was exerted as described before [17, 18]. Isolated ECs or cryosections of aortas were fixated with 4% paraformaldehyde and then permeabilized with 0.2% TritonX-100. Unspecific binding sites were blocked with 5% donkey serum (Jackson ImmunoResearch, Suffolk, UK) for 30 min. Then, cells or sections were incubated with primary antibodies for 3 h: anti-alpha smooth muscle actin (1:400, anti-ASMAC, A5228, Sigma-Aldrich), anti-CD31 (1:800, 550274, BD Biosciences), anti-HOXC10 (1:100, Thermo Fisher, 12025–1-Ap), anti-CDH11 (1:100, 71-7600 Invitrogen), anti-C7 (1:100, PA5-120912, Invitrogen) and anti-GREM2 (1:100, 13892-1-AP, Proteintech), anti-CD45 (1:800, 05-1416, Merck) and anti-FLK-1 (1:100, ab2349, Abcam). After that, Cy3- or Cy5-labeled anti-rat/rabbit secondary antibodies (Jackson ImmunoResearch) were applied for 1 h. Nuclei were stained with hoechst (1:1000, Sigma-Aldrich). Embedding of the cells and sections was performed with Aqua-Poly/Mount (Polyscience, Warrington, USA) and pictures were taken with an AxioObserverZ1 microscope equipped with an apotome module (Zeiss, Oberkochen, Germany). Alternatively, diaminobenzidine (DAB) staining was performed using Vectastain Elite kits and DAB (Biomol, Germany), embedding was performed with Entellan and pictures were taken by an ECLIPSE Ci-L microscope (Nikon, Düsseldorf, Germany). For DAB stainings two sections of each aortic segment dervied from two aortas were analyzed, analysis was un-blinded.

### Quantification of elastin breaks

For quantification of elastin breaks autofluorescence of elastin in fluorescence pictures was used. Elastin breaks were counted manually at 40 × magnification on pictures of 3 non-overlapping areas of the aortic media per segment and per mouse. Each data point represents the mean value of one segment and mouse.

### RNA-seq analysis

**Fig. 1:**
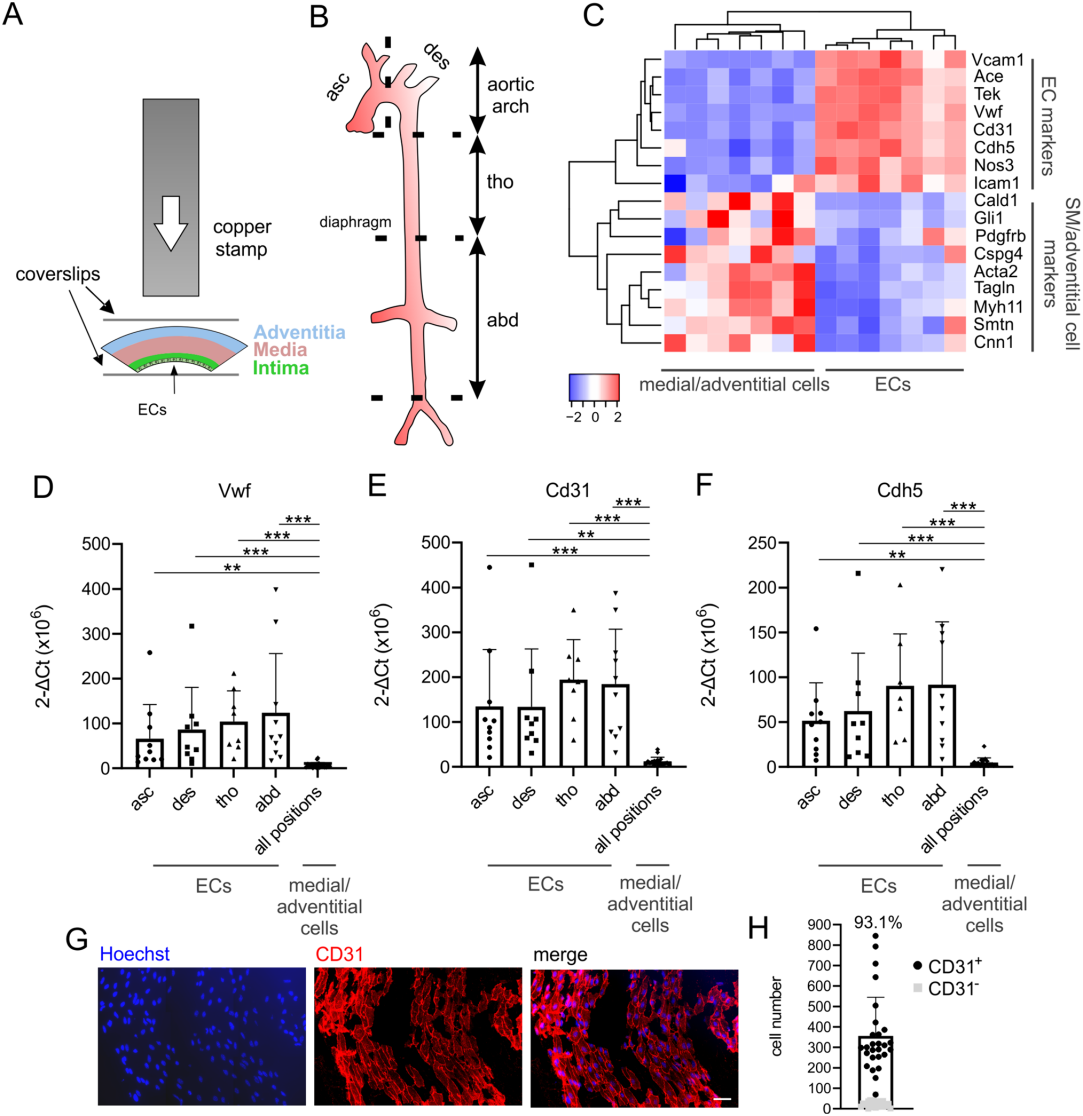
Modified “Häutchen method” for high enrichment of ECs from distinct locations of mouse aorta. **A** Schematic diagram of the modified “Häutchen method”.** B** Schematic diagram of different aortic segments (asc: ascending arch, des: descending arch, tho: thoracic aorta, abd: abdominal aorta). **C** Heatmap of EC and SMC markers in ECs and medial/adventitial cells from aorta isolated with the modified “Häutchen method”, for this experiment thoracic and abdominal ECs or adventitial cells were pooled. **D**–**F** mRNA expression of EC-specific markers Vwf (**C**), Cd31 (**D**) and Cdh5 (**E**) in ECs and adventitial cells isolated from different aortic segments using qPCR. **G** Immunostaining of ECs isolated with the modified “Häutchen method” using anti-CD31 antibody (red), nuclei are labeled with Hoechst (blue), scale bar: 50 µm. **H** Quantification of CD31^+^ cells by counting. **D**–**F** Kruskal–Wallis test, Dunn`s post hoc test, **p < 0.01, ***p < 0.001

RNA-seq analysis was performed as describe before [16]. For library preparation, the Trio RNA-Seq Library Preparation kit (TECAN, Männedorf, Switzerland) was used. Five PCR cycles were applied for library amplification and libraries with an average fragment size of 317 bp were sequenced on a NextSeq 500 in paired-end mode (65 bp, NextSeq 2000), data for Fig. 1C were sequenced in single-end mode at the GeneCore sequencing service of the EMBL (Heidelberg, Germany). For bioinformatic analysis, we used the Galaxy platform (Freiburg Galaxy Project). RNA sequencing reads were mapped using RNA STAR followed by counting reads per gene by using featureCounts. As an additional quality control step the purity of ECs in the respective sample was determined by analysing expression levels of the classical EC marker genes Cd31, VwF and Cdh5. The normalized counts of these 3 marker genes were added up for each sample and compared with EC marker expression in adventitial samples from 2 control animals. Only samples with EC marker expression of > twofold of the mean EC marker expression in adventitial samples were included in the analysis. In the remaining samples, differentially expressed genes were identified by DESeq2. For data visualization, normalization, and cluster analysis heatmap2 and Volcano plot (Freiburg Galaxy Project) was used. Gene ontology analysis of the up- and downregulated genes was performed with ClueGO using the GO-term database with the sub-ontologies “biological processes, cellular-component and molecular function”.

### Single-cell force spectroscopy by atomic force microscopy (AFM)

The nanomechanical properties of the endothelial actin cortex were determined by using the Atomic Force Microscopy (AFM)-based single-cell force spectroscopy (Nanowizard4, JPK, Berlin, Germany) as described before [19]. Briefly, a triangular cantilever (Novascan Technologies, Boone, North Carolina, United States) with a mounted spherical tip (diameter 10 μm) and a nominal spring constant of 30 pN/nm indents the ECs on the aortic patch with a loading force of 3 nN. The reflection of a laser beam is used to quantify the cantilever deflection. By knowing the deflection sensitivity, the cantilever force and the piezo displacement, the stiffness (in pN/nm) of the cell cortex can be calculated from the resulting force-distance curves using the Protein Unfolding and Nano-Indentation Analysis Software PUNIAS 3D version 1.0 release 2.2 (http://punias.voila.net).

Harvesting and preparation of aortas in order to analyze the cortical stiffness of single mouse aortic ex vivo EC by AFM were carried out as described before[20]. Briefly, aortas from male C57BL/6 (age 11–12 weeks) were freed from surrounding tissue. Small patches of the whole aorta (≈ 4 mm^2^) were attached on glass coverslips with Cell-Tak® (BD Biosciences, Bedford, MA, USA), with the endothelial surface facing upwards. After preparation, the aortic patches were cultured until the next day for AFM measurements in minimal essential medium (MEM; Invitrogen Corp., La Jolla, CA, USA) supplemented with 10% fetal calf serum (FCS; PAA Laboratories, Pasching, Austria), 1% MEM vitamins (Invitrogen), 1% MEM nonessential amino acids (Invitrogen) and 1% Penicillin/Streptomycin (100 U/ml; 100 mg/ml) under standardized cell culture conditions.

### Statistical analysis

Statistical analysis was performed using Prism 8 (GraphPad, San Diego, USA). Data are presented as mean ± SD. Each data point represents a biological replicate. For comparison of differences between more than two groups with normal distribution One way ANOVA with Tukey’s post hoc test was used, in case of non- normal distribution of values Kruskal–Wallis test was applied. For comparisons of differences between more than two groups with data of unequal variances Welch’s ANOVA with Dunnett’s post hoc test was used. P values < 0.05 were considered significant.

## Results

### Site-specific isolation of ECs from the aorta of healthy C57BL/6 mice using the modified “Häutchen method”

We first analyzed ECs from distinct locations along the aortic tree of healthy C57BL/6 mouse aortas. To this aim ECs from the intima and cells from the media/adventitia were isolated from aortic sections of the ascending and descending arch as well as the thoracic and abdominal part of the straight segments of the aorta. Therefore, we used the modified “Häutchen method” (Fig. 1A,B) that enables to separate superficial cell layers from multicellular tissues by their adherence to glass coverslips. Isolation of RNA and analysis of bulk RNA-seq data proved prominent enrichment of aortic ECs and of medial/adventitial cells by this method as correct clustering of samples derived from the endothelium and the media/adventitia was found in a heatmap of selected EC and smooth muscle (SM)/adventitial cell markers (Fig. 1C). This was further confirmed by qPCR of new samples revealing strongly enhanced expression of the prototypic EC markers von Willebrand factor (VwF), PECAM (Cd31) and VE-cadherin (Cdh5) in cells harvested from the endothelial, but not from the medial/adventitial side of the aorta (Fig. 1D–F). We also quantified the number of total cells and ECs obtained with the modified “Häutchen method” by applying Hoechst as well as CD31 staining and counting (Fig. 1G). We found 356 ± 189 (n = 27) ECs from each of the different aortic locations adherent on single cover slips (Fig. 1H); the number of ECs derived from different segments of the aorta was overall similar (260 to 408 cells, p > 0.05). Importantly, the vast majority (91–95%) of isolated cells from the endothelial side of all aortic segments was CD31^+^. Thus, the modified “Häutchen method” enables strong enrichment of ECs derived from specific locations of the aortic tree.

### Differential gene expression of ECs derived from distinct sites of healthy aorta

**Fig. 2:**
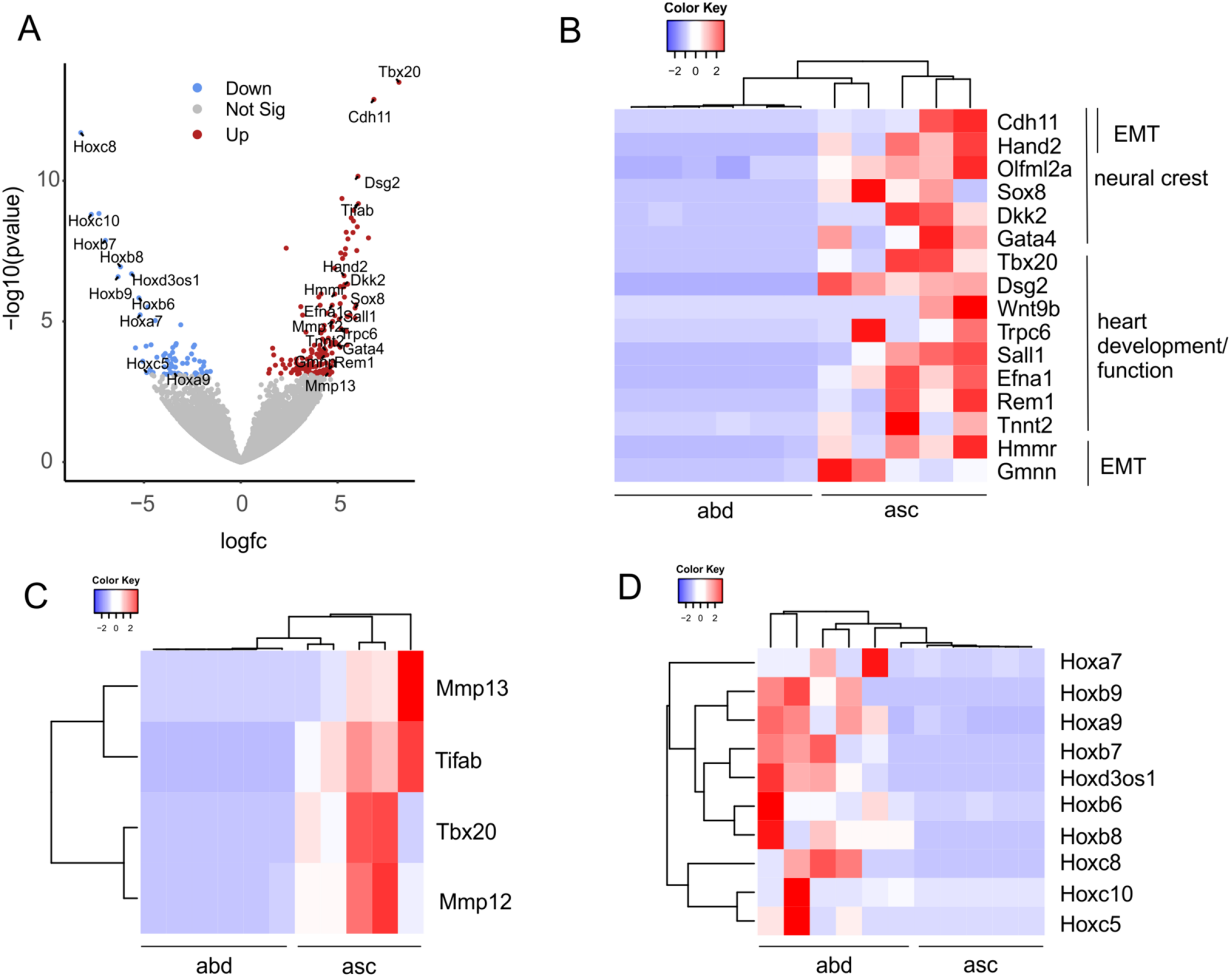
Differential expression of marker genes for neural crest/heart development or embryonic patterning in aortic ECs from the ascending arch (asc) vs the abdominal aorta (abd). **A** Volcano plot of up- and downregulated genes in the ascending arch vs the abdominal aorta. **B** Heatmap of DEGs related to neural crest or heart development/function and epithelial to mesenchymal transition (EMT) in the respective samples. **C** Heatmap of DEGs related to arterial/aortic aneurysm formation. **D** Heatmap of DEGs related to the Hox gene family

Then we compared the number of differentially expressed genes (DEGs) in ECs derived from different regions of healthy C57BL/6 aortas and found that it increased with greater distance between the respective aortic segments: 68 DEGs in the ascending vs descending arch, 154 in the ascending arch vs thoracic aorta, 222 in the ascending arch vs abdominal aorta (Tables S1–3). The analysis of ECs from the most distant regions, namely the ascending arch (n = 5) and the abdominal aorta (n = 6), reveals that genetic signatures were strongly determined by their developmental origins: In the ascending aorta ECs displayed upregulation of (cardiac) neural crest markers and regulators[21–26] (Fig. 2A, B). Likewise, we found upregulation of genes involved in heart development/function and valve morphogenesis [22, 27–37] (Fig. 2A, B) as well as epithelial to mesenchymal transition (EMT) [38–41] (Fig. 2A, B). Interestingly, we also detected an upregulation of genes that have been linked to aneurysm formation in earlier studies [42–45] (Fig. 2C). In ECs from the abdominal aorta, we found an upregulation of various homeobox (Hox) genes (Hox 5–10) that are known to contribute to the development of this part of the aorta [46] (Fig. 2A, D), further underscoring the validity of our approach. Regionally restricted Hox gene expression reflects embryonic patterning during aortic development and is considered as a sign for the positional identity of the cells also in the adult [47]. While most of these developmental markers had been previously identified in smooth muscle cells we can show by immunofluorescence staining that they are expressed in both, in ASMAC^+^ smooth muscle cells and CD31^+^ ECs of the aorta further proving the validity of our approach (Figure S1A–D).

**Fig. 3:**
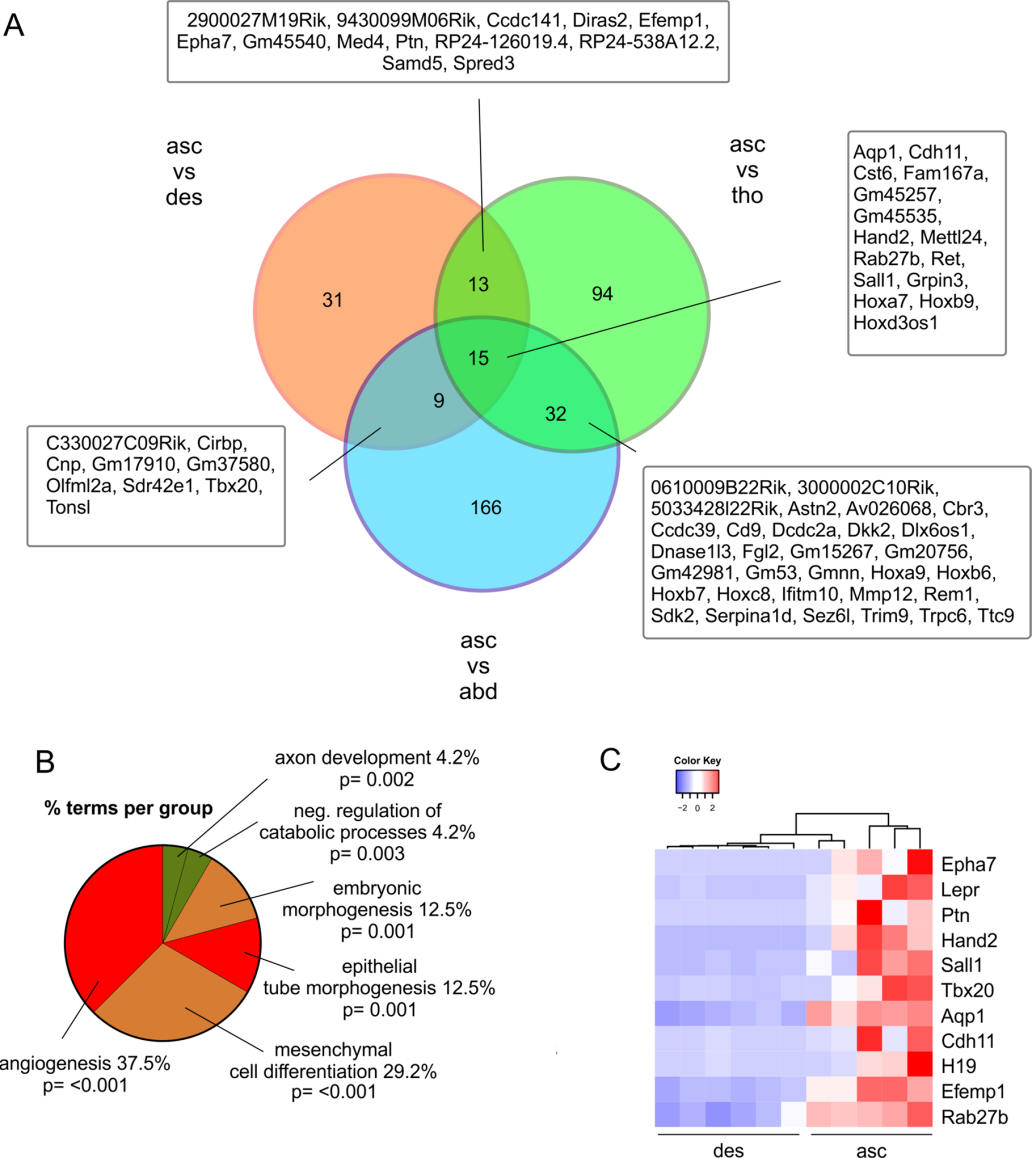
Differential expression of pro-angiogenetic genes in aortic ECs derived from the ascending (asc) vs the descensing (des) arch.** A** Venn diagram of DEGs in ECs from ascending arch vs all other aortic segments. **B** GO analysis of the category “biological processes” of the DEGs in ECs from ascending vs dessending arch. **C** Heatmap of pro-angiogenic DEGs in the respective samples

Next, we compared gene expression of healthy ECs derived from AA predilection sites with respective control segments. Because the aortic root and the ascending arch are typical localizations for aneurysm formation, first, we compared gene expression of ECs derived from this segment (n = 5) with all other aortic segments and identified 15 common DEGs (Fig. 3A) that are most typical for the ascending arch of aorta. We again found upregulated genes displaying neural crest/heart development markers (Cdh11, Hand2, Sall1) and downregulated genes characteristic for distal aorta development (Hoxa7, Hoxb9) (Fig. 3A, Table 1). Interestingly, the upregulated genes in ECs from the ascending arch also reflect a pro-angiogenic signature (Cdh11, Hand2, Sall1, Aqp1, Rab27b) (Fig. 3A, Table 1). We then compared the ECs from the ascending arch (n = 5) with the adjacent control segment of the descending arch (n = 6) and identified 39 up- and 29 downregulated genes. Gene ontology (GO) analysis revealed differential regulation of genes related to mesenchymal cell differentiation and embryonic morphogenesis (Fig. 3B), the highest number of DEGs, however, could be attributed to the categories of angiogenesis and epithelial tube morphogenesis (Fig. 3B). In particular, the upregulated genes are known to mediate pro-angiogenic effects either directly (Epha7 [42], Lepr [48], Ptn [49], Hand2 [50], Sall1 [51], Tbx20 [52], Aqp1 [53]) or indirectly (Cdh11 [54], H19 [55], Efemp1 [56], Rab27b [57]) (Fig. 3C). Because the typical EC marker expression (Vwf, Cd31, Cdh5) was similar in ECs from the ascending and descending arch it can be excluded that the differential expression of pro-angiogenic genes was due to different EC purities in the samples. Thus, ECs from the AA predilection site of the ascending arch are characterized by upregulation of pro-angiogenic genes.

**Table 1:** Common up- and downregulated DEGs in asc

Gene symbol	Comparison	log2(FC)	P adj		Function
*Up*					Neural crest Heart Development Pro-angiogenic
Cdh11	asc vs des	3.80	0.001		
	asc vs tho	4.36	< 0.001		
	asc vs. abd	6.87	< 0.001		
Hand2	asc vs des	5.60	< 0.001		
	asc vs tho	5.82	< 0.001		
	asc vs. abd	5.33	< 0.001		
Sall1	asc vs des	3.71	0.003		
	asc vs tho	5.32	< 0.001		
	asc vs. abd	5.10	0.002		
Aqp	asc vs des	2.82	< 0.001		Pro angiogenic
	asc vs tho	2.98	< 0.001		
	asc vs. abd	3.10	0.001		
Rab27b	asc vs des	2.05	0.007		Pro angiogenic
	asc vs tho	2.12	< 0.001		
	asc vs. abd	2.07	0.049		
*Down*					
Hox a7	asc vs des	−4.34	< 0.001		Distal aorta Development
	asc vs tho	−5.40	< 0.001		
	asc vs. abd	−5.20	0.002		
Hox b9	asc vs des	−3.44	0.041		
	asc vs tho	−7.18	< 0.001		
	asc vs. abd	−6.33	< 0.001		

*DEGs* differentially regulated genes, *asc* ascending arch, *Dds* descending arch, *tho* thoracic aorta, *abd* abdominal aorta

**Fig. 4:**
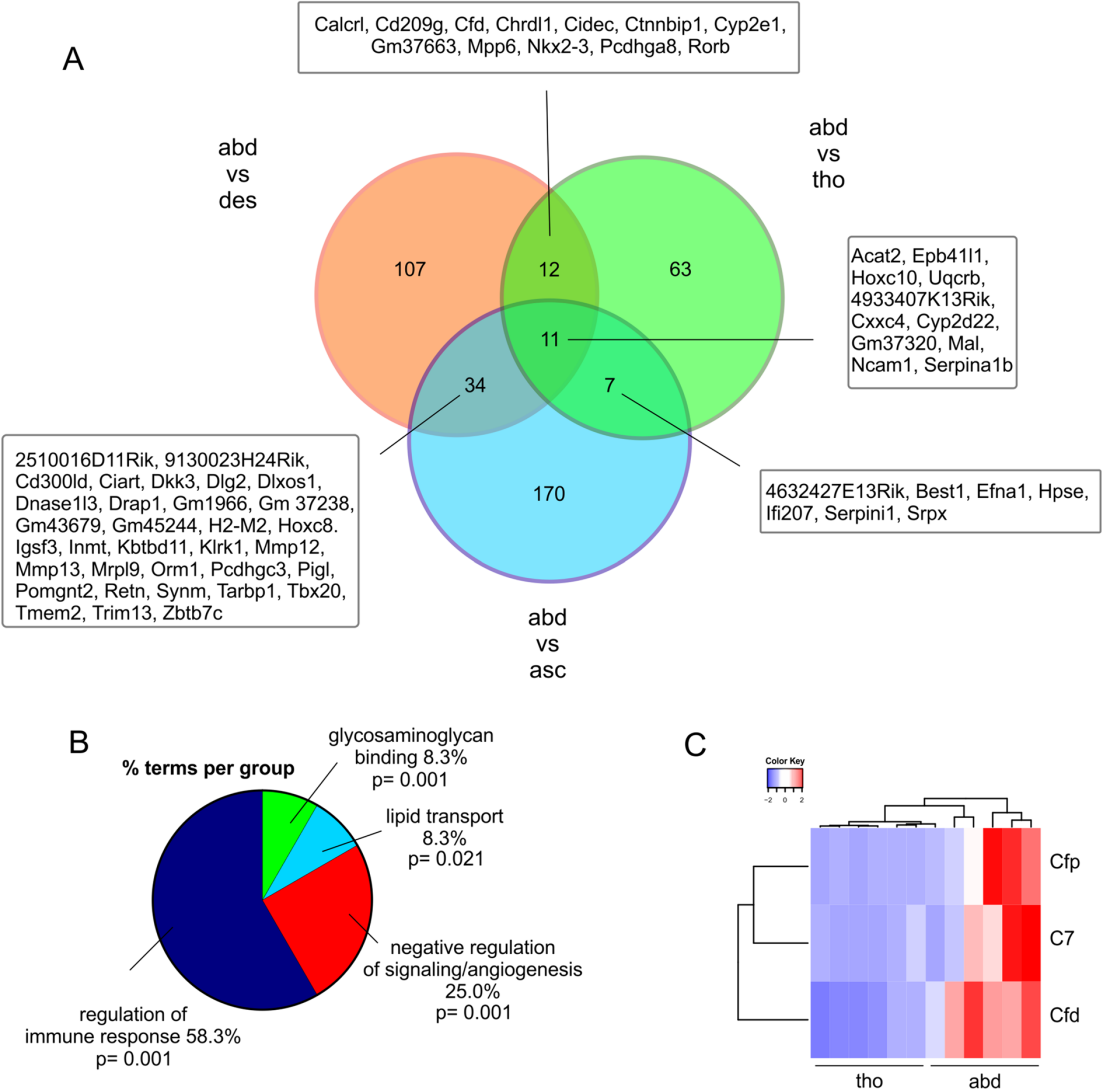
Differential expression of ECM-related, pro-angiogenic and pro-inflammatory genes in aortic ECs derived from the abdominal (abd) vs the thoracic (tho) aorta. **A** Venn diagram of DEGs in ECs from abd aorta vs all  other aortic segments. **B** GO analysis of the category “biological processes” of the DEGs in ECs from abdominal vs thoracic aorta. **C** Heatmap of DEGs related to the complement system in the respective samples

Next, we compared gene expression of ECs from the other typical predilection site for aneurysm development, the abdominal aorta (n = 6), with all the other segments and found 11 common DEGs (Fig. 4A). The upregulated genes are representative of cholesterol and fatty acid metabolism (acat2) [58], cell adhesion and migration (Epb41l1) [59] and angiogenesis (Hoxc10 [60], Uqcrb [61]) (Fig. 4A, Table 2). When we compared ECs from the abdominal segment (n = 6) with those of the adjacent control segment, the thoracic aorta (n = 6), we found 57 up- and 36 downregulated genes. GO analysis revealed differential regulation of genes related to ECM-related glycosaminoglycan binding, lipid transport, and negative regulation of signaling (Fig. 4B). In the latter category inhibitors of Wnt, BMP, and EGF signaling (Ctnnbip1 [62], Cxxc4 [63], Grem2 [64], Errfi1 [65]) were detected, but it also contained upregulated pro-angiogenic genes (Chrdl1 [66], Dcn [67], Ecm1 [68], Igf1 [69], Fig. 4B, Table 3). This revealed that also in ECs of the abdominal aorta a genetic signature of altered angiogenesis was found. The category with the most DEGs comparing ECs from abdominal and thoracic aorta, was related to regulation of the immune response with the majority of these genes being upregulated (Table 3) [64, 70–81]. Interestingly, we found members of the complement system to be upregulated that was demonstrated to be involved in atherosclerosis and particularly in AAA before [82, 83] (Fig. 4C). Thus, ECs from the AA predilection site of abdominal aorta are characterized by differential expression of markers for ECM binding, angiogenesis, and immune response.

**Table 2:** Common upregulated DEGs in abd

Gene symbol	Comparison	log2(FC)	P adj	Function
Acat2	abs vs asc	2.39	0.012	Cholesterol and fatty acid metabolism
	abd vs des	2.37	< 0.001	
	abd vs tho	2.79	0.026	
Epb41I1	abs vs asc	2.54	0.020	Cell adhesion and migration
	abd vs des	2.50	< 0.001	
	abd vs tho	3.16	0.025	
Hoxc 10	abs vs asc	7.72	< 0.001	Pro-angiogenic
	abd vs des	8.04	< 0.001	
	abd vs tho	8.12	< 0.001	
Uqcrb	abs vs asc	2.05	0.044	Pro-angiogenic
	abd vs des	2.37	< 0.001	
	abd vs tho	1.92	0.042	

*DEGs* differentially regulated genes, *asc* ascending arch, *des* descending arch, *tho* thoracic aorta, *abd* abdominal aorta

**Table 3:** DEGs related to angiogenesis and immune response in abd vs tho

Gene symbol	log2(FC)	P adj	Genesymbol	log2(FC)	P adj
Angiogenesis			Immune response		
*Up*			*Up*		
Chrdl1	3.17	0.036	Grem2	7.75	< 0.001
lgf1	2.86	0.039	Nfil3	5.13	0.004
Dcn	2.58	0.043	Slamf1	4.93	0.024
Ecm1	1.85	0.049	Cfp	3.76	< 0.001
			C7	3.46	0.019
*Down*			H2-Ab1	2.84	0.027
Twist1	−3.21	0.023	Ifi207	2.75	0.036
			Cfd	2.54	< 0.001
			Ighm	2.30	0.048
			Ecm1	1.85	0.049
			*Down*		
			Twist1	−3.21	0.023
			Cd47	−1.13	0.031

*DEGs* differentially regulated genes, *tho* thoracic aorta, *abd* abdominal aorta

**Fig. 5:**
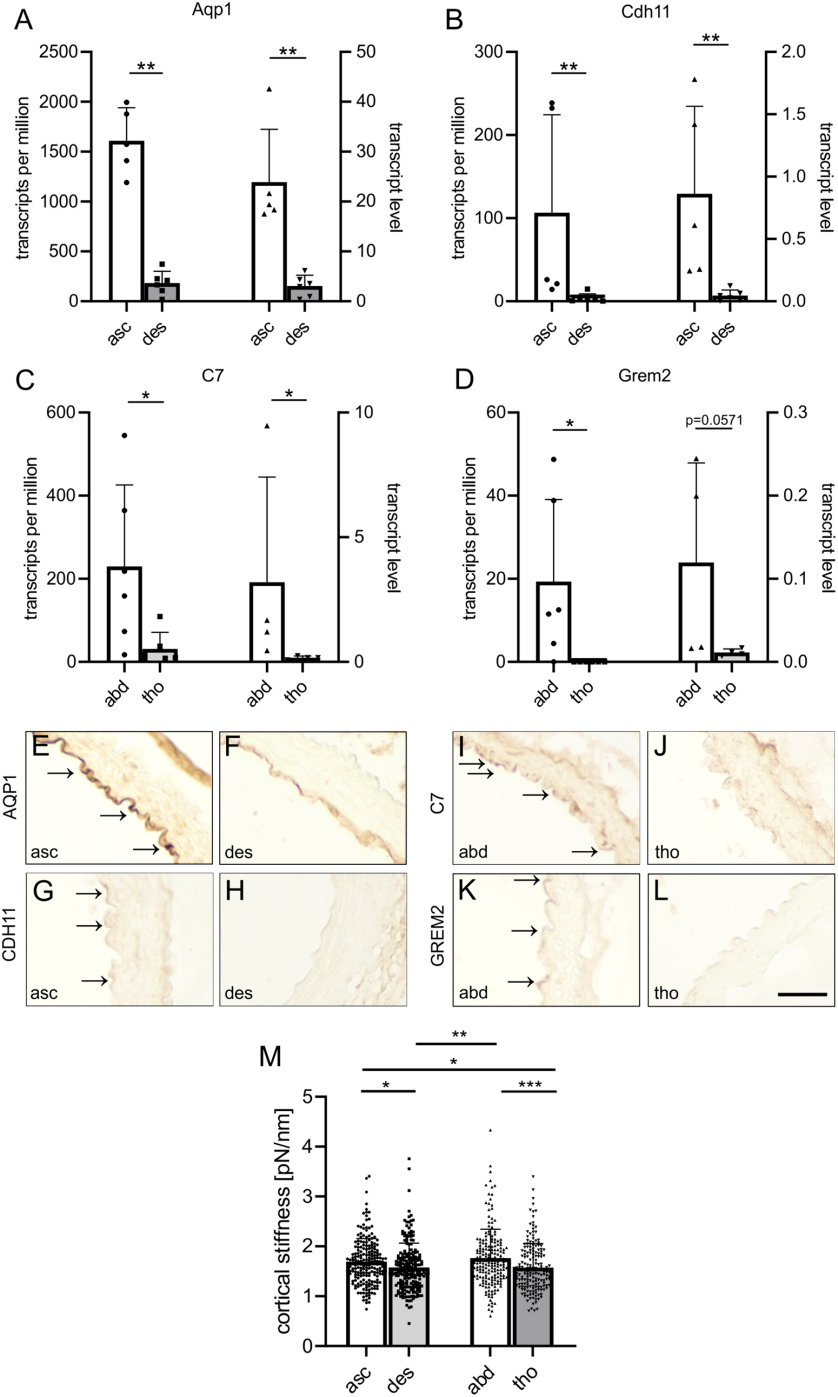
Analysis of gene expression by dPCR and protein expression by DAB staining, mechanical properties of the endothelium.** A**–**D** Comparison of gene expression by RNA-seq (left) and dPCR (right) of strongly expressed genes in the endothelium of the ascending arch (asc): Aqp1 (**A**) and Cdh11 (**B**) as well as of the abdominal aorta (abd): C7 (**C**) and Grem2 (**D**). **E**–**L** DAB staining of AQP1 (**E**, **F**), CDH11 (**G**, **H**), C7 (**I**, **J**) and GREM2 (**K**, **L**) in the endothelium of the aortic segments indicated, arrows point at staining in ECs, scale bar: 50 µm. **M** Assessment of cortical stiffness of ECs from the different segments of healthy mouse aortas, N = 4. **A**–**D** unpaired student’s t-test, **M** Kruskal–Wallis test, Dunn’s post hoc test, *p < 0.05, **p < 0.01, ***p < 0.001

In order to confirm the results of the RNA-seq analysis we used the same samples and performed dPCR of strongly expressed genes regulated in ECs of the ascending arch and abdominal aorta. Our results showed that the expression pattern of exemplary genes characteristic for ECs from the ascending arch (Aqp1 and Cdh11), and for the abdominal aorta (C7 and Grem2) was very similar when comparing values obtained with dPCR (Fig. 5A–D right bars and axis) or RNA-seq (Fig. 5A–D, left bars and axis). RNA-seq data were further confirmed by dPCR analysis of more genes in newly isolated EC samples from the ascending and descending arch (Efemp1, Cd55, Ptn, Hand2, Figure S1 E–H) and from the abdominal and thoracic aorta (Cfd, Cidec, Hoxc10, Figure S1 I–K).

We also performed immunohistochemistry of aortic sections and using fluorecence stainings we found co-localization of AQP1 (Figure S1 L) and CDH11 (Figure S1 M) as well as C7 (Figure S1 N) and GREM2 (Figure S1 O) with CD31^+^ ECs in the ascending and abdominal segments of the aorta, respectively, confirming protein expression of these regulated genes in ECs. We also assessed differences in protein expression in ECs from adjacent aortic segments using semi-quantitative DAB stainings. These revealed stronger signals for AQP1 (Fig. 5E, F) and CDH11 (Fig. 5G, H) in ECs from the ascending vs the descending aortic arch and for C7 (Fig. 5I, J) and GREM2 (Fig. 5K, L) in ECs from the abdominal vs the thoracic aorta.

Besides gene expression, we also investigated if the mechanical properties of ECs from aneurysm predilection sites differed from the respective control regions by measuring the cortical stiffness of single ECs ex vivo by atomic force microscopy (AFM)-based single-cell force spectroscopy. Enhanced aortic stiffness has been reported to reflect a susceptibility to aneurysm formation [84] and stiffening of ECs is an early sign of pathological changes as it is known to correlate with endothelial dysfunction and to increase during ageing [85]. In *en face* preparations of healthy mouse aortas we found elevated cortical stiffness of single ECs in segments of the ascending (1.7 ± 0.5 pN/nm, N = 4, n = 197) compared to the descending (1.6 ± 0.5 pN/nm, N = 4, n = 204, p = 0.024) arch, and similar results were obtained in aortic segments from the abdominal (1.8 ± 0.6 pN/nm, N = 4, n = 188) vs the thoracic (1.6 ± 0.5 pN/nm, N = 4, n = 182, p = 0.0008) aorta (Fig. 5M). As control, we determined stiffness in different locations around the circumference of the ascending or descending aorta and found that it was very similar (asc: p = 0.4, des: p = 0.7). Thus, the endothelium from aneurysm predilection sites displays increased cortical stiffness already in healthy aortas providing additional evidence that the endothelium is altered at aneurysm predilection sites.

### Analysis of gene expression changes in ECs from ascending arch and abdominal aorta in the AngII ApoE^−/−^ aneurysm model

**Fig. 6:**
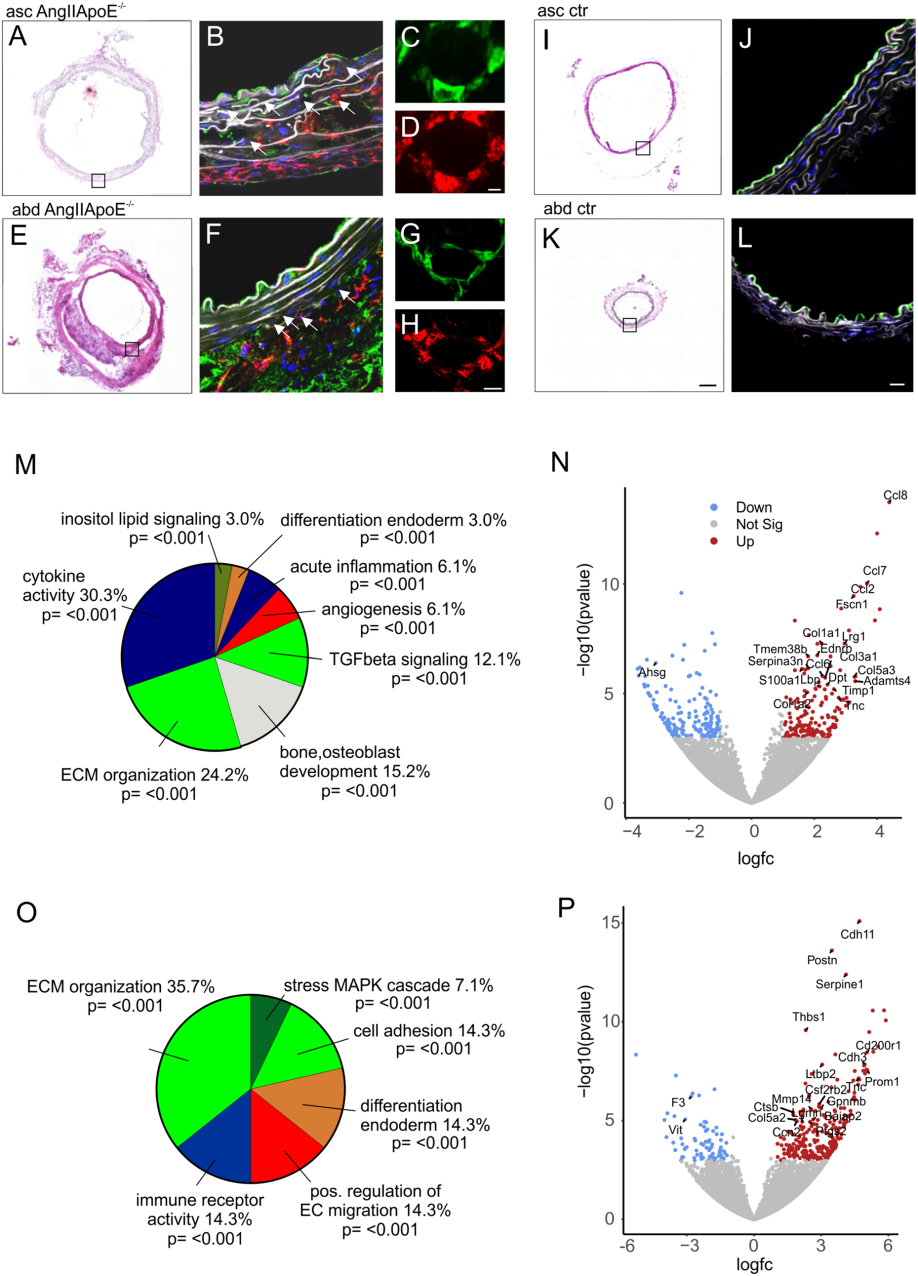
Differential expression of genes related to ECM organization, angiogenesis and inflammation in ECs from the ascending arch (asc) and abdominal aorta (abd) in the AngII ApoE^−/−^ model. **A**–**L** H&E stainings (**A**, **E**, **I**, **K**) and immunofluorescence stainings (**B**–**D**, **F**–**H**, **J**, **L**) of aneurysms in the ascending arch (**A**–**D**) or abdominal aorta (**E**–**H**) of the AngII ApoE^−/−^ model (d28) vs sham controls of the ascending arch (**I**, **J**) and the abdominal aorta (**K**, **L**), arrows indicate elastin breaks, green = CD31, red = CD45 (**B**, **F**, **J**, **L**) or FLK1 (**D**, **H**), white = autofluorescence, blue = hoechst. **M** GO analysis of the category “biological processes” of the DEGs in ECs from the ascending arch of AngII ApoE^−/−^ mice (d14) vs sham mice. **N** Volcano plot of up- and downregulated genes in the ascending arch of AngII ApoE^−/−^ mice vs sham mice. **O** GO analysis of the category “biological processes” of the DEGs in ECs from the abdominal aorta of AngII ApoE^−/−^ mice (d14) vs sham mice. **P** Volcano plot of up- and downregulated genes in the abdominal aorta of AngII ApoE^−/−^ mice vs sham mice, scale bars: 200 µm (**A**, **E**, **I**, **K**), 20 µm (**B**, **F**, **J**, **L**), 5 µm (**G**, **H**), 2 µm (**C**, **D**)

Next, we analyzed aneurysms in the ascending arch and abdominal aorta derived from the AngII ApoE^−/−^ model at d14 or d28 and compared them with the same sites in sham animals (ApoE^−/−^ mice without AngII application). In the vast majority of the AngII ApoE^−/−^ mice we found aneurysms in the respective locations (7/9 asc, 8/9 abd) that could also be detected by ultrasound imaging. Quantitative analysis revealed elevated aortic diameters in AngII ApoE^−/−^ mice vs shams at d14 (Figure S2 A, B). H&E stainings of aortic sections confirmed an increased diameter in the ascending arch (Fig. 6A) and in the abdominal aorta (Fig. 6E) compared to control sham animals (Fig. 6I, K) consistent with aneurysm formation. This was also underscored by elastin breaks due to altered ECM organization in the aortic wall of both sites (arrows, Fig. 6B,F; Figure S2 C). These changes were accompanied by CD31^+^ cells (green) in the aortic wall (Fig. 6B ,F) as well as FLK-1^+^ (red) CD31^+^ (green) vascular structures (Fig. 6C, D, G, H) most likely highlighting vasa vasorum and by CD45^+^ cells (red) indicating inflammation (Fig. 6B, F), all these changes were largely absent in the respective aortic segments of sham animals (Fig. 6J, L). These typical pathophysiological alterations mirrored the transcriptome profile of the endothelium at aneurysm predilection sites in healthy aortas. Thus, we wondered if these genetic changes can also be found in the endothelium of aortas with aneurysm. First, we compared ECs from the ascending arch or the abdominal aorta of sham (ApoE^−/−^ with western diet) with WT animals and detected a pro-inflammatory signature (Fig. S3 A, B), which is in accordance with endothelial alterations in the ApoE^−/−^ model. RNA-seq analysis of ECs isolated from manifest aneurysms of the ascending arch of the AngII ApoE^−/−^ mice vs ECs from the same segment of sham animals confirmed upregulation of DEGs related to the categories of ECM organization, TGFbeta signaling, angiogenesis and cytokine activity as well as acute inflammation (Fig. 6M). The strongest upregulated genes belonged to the pro-inflammatory chemokine family (Ccl2, Ccl7, Ccl8) (Fig. 6N). When we compared the gene expression pattern of ECs from aneurysms of the abdominal segment of AngII ApoE^−/−^ mice with shams we found very similar categories to be regulated with ECM organization, cell adhesion, positive regulation of EC migration and immune receptor activity (Fig. 6O). The most upregulated genes link ECM remodeling, angiogenesis and inflammation to aneurysm formation such as Cdh11 [86], Postn [87], Serpine1 [88, 89] and Thbs [90, 91] (Fig. 6P). Interestingly, we found two distinct genes (Abcb1a, Cd53) that are upregulated in ECs from the healthy abdominal WT aorta as well as in ECs from abdominal aneurysms. Taken together, the gene expression pattern of ECs derived from the different sites of aneurysm formation vs sham animals shows some similarity with that found in predilection sites of healthy aortas when compared to the adjacent control regions. Thus, the heterogeneity of EC gene expression signatures in healthy mice indicates the location and pathophysiological alterations of aortic aneurysm formation**.**

## Discussion

Aortic aneurysms develop at specific predilection sites, namely the aortic arch and the abdominal aorta. So far it is unclear if this is solely due to different hemodynamic forces or if also intrinsic differences of the vascular wall play a role. In fact, earlier work from Haimovici et al. has provided intriguing experimental evidence for the genetic determination of aortic disease. They transplanted canine abdominal aortic grafts that are prone to atherosclerosis into the thoracic aorta or the jugular vein of dogs that were fed an atherogenic diet. The grafts were found to develop severe lesions in the new location while local vessels were much less affected. This suggested that the susceptibility to aortic disease is determined by tissue properties rather than hemodynamic flow conditions [92, 93].

These site-specific intrinsic differences of the aorta may be related to their different developmental origin as SMCs from different parts of aorta are derived from different embryonic tissues [94, 95] and this correlates with the susceptibility of the cells to calcification and aortic disease [96, 97]. We demonstrate that the developmental origins of the different aortic segments are preserved in the RNA signatures of healthy mouse ECs of the aorta, as we detected an upregulation of either neural crest-related genes in ECs from the ascending arch or of various Hox genes in ECs from the abdominal part. Interestingly, these developmental genes were reported to contribute to aortic aneurysm of the ascending [98, 99], and abdominal [100] AA formation and dissection in humans. Most of these studies focused on whole aortic tissue or SMCs. Our data on ECs demonstrate that their gene expression patterns are very similar to adjacent SMCs (e.g., neural crest-specific genes, Hox genes). Interestingly, the concept that ECs from the brain, lung and heart express genes that are also found in surrounding cells and tissues has been proposed in the past and suggests an organ-specific plasticity of ECs [101]. EC heterogeneity in various organs of mouse [9, 102] but also within the aorta has previously been detected by scRNA-seq analyses and enabled the identification of 2 or 3 distinct aortic EC populations [103–106]. However only He et al. could assign EC populations to distinct aortic segments. Interestingly, this group also characterized aortic ECs from mice exposed to high fat/salt/glucose conditions and, similar to our data from ECs of AA, found the appearance of ECs with high Serpine1 expression [105].

Besides changes in the gene expression pattern recent studies emphasized the important role of altered EC function in aneurysm formation as endothelial dysfunction, eNOS uncoupling and defective EC barrier function were found to be involved in the pathophysiology of the disease[107–109]. Our data also reveal early signs for endothelial dysfunction in ECs of healthy animals, as we detected elevated endothelial stiffness. Accordingly, some of the differentially expressed genes we found in ECs from the ascending arch and the abdominal part of healthy aortas suggest altered mechanical properties of the cells. For instance, Cdh11 [110] and Dcn [111] were reported to regulate collagen and elastin synthesis, while Hand2 has an impact on the cytoskeleton [112], all processes affecting the mechanical properties of tissues. Moreover, Aqp1 was claimed to be directly involved in aortic stiffening in diabetes [113]. Our finding of enhanced cortical stiffness at aneurysm predilection sites is consistent with earlier reports highlighting segmental aortic stiffening as an early pathomechanism evoking aneurysm formation in mouse [114] and humans [115, 116]. Even though aneurysm formation only develops in the ApoE model with AngII infusion also ECs from our sham mice (ApoE^−/−^ with western diet) showed an altered pro-inflammatory gene expression pattern compared to WT animals. This is similar to a previous study where gene expression of whole aortas from ApoE^−/−^ vs WT animals were compared [117] and corresponds to the well-known development of atherosclerosis in this mouse model. Yet, the additional AngII application triggers aneurysm development and further induces gene expression related to ECM remodeling, angiogenesis and inflammation.

The gene expression pattern we found in ECs from aneurysms of the AngII ApoE^−/−^ model fits very well to reported pathophysiological mechanisms of aneurysm formation that have been identified in whole aortic tissues of aneurysms: We detected changes of ECM-related genes such as collagens [118], metalloproteinases and proteoglycans/glycoproteins [119] and a dysregulation of lysyloxidase (LOX) expression [120]. In addition, we found regulation of angiogenetic modulators such as pro-angiogenic GATA6 [121], leucine-rich alpha-2- glycoprotein 1 (LRG1) as a regulator of pathogenic angiogenesis [122] and osteonectin/SPARC that can regulate EC shape and barrier function [123]. Finally, there was also increased expression of pro-inflammatory endothelial chemokines (e.g., Ccl2, Il6) [124], known to potentiate inflammatory processes and to be involved in aneurysm pathophysiology [125, 126]. Nevertheless there are also some limitations of the study, namely a relatively low number of cells that can be isolated and therefore limited material for PCR or protein analysis. Future studies taking advantage of spatial transcriptomics or multiplexed error-robust fluorescence in situ hybridization (MERFISH) may be able to further improve the spatial resolution within the aorta and of ECs [127] and provide more insights into the site specific heterogeneity of ECs.

Thus, we have detected genetic signatures in ECs from aneurysm predilection sites of healthy mouse aortas that are not identical but correlate with changes found in manifest aneurysms suggesting that these define the site and pathophysiological alterations of aneurysm formation in aortic disease.

## Supplementary Information

Below is the link to the electronic supplementary material.

Supplementary file1 (DOCX 1632 KB)

## Data Availability

All data associated with this study are present in the paper or the supplementary materials. All sequencing data sets reported in this manuscript are deposited in the Short Read Archive at the National Center for Biotechnology Information under the BioProject ID PRJNA1105313. Additional data that support the findings of this study are available from the corresponding author upon request. Source data are provided with this paper.
