# Supplementary material for: Site-specific genetic and functional signatures of aortic endothelial cells at aneurysm predilection sites in healthy and AngII ApoE−/− mice
Source: Angiogenesis. 2024 Jul 4;27(4):719–38. doi: 10.1007/s10456-024-09933-9 (PMC11564227; doi:10.1007/s10456-024-09933-9)
Supplement: Supplementary file 1 — Supplementary file1 (DOCX 1632 KB) [file 10456_2024_9933_MOESM1_ESM.docx]

Supplemental Material

**Site-specific genetic and functional signatures of aortic endothelial cells at aneurysm predilection sites in healthy and AngII ApoE^-/-^ mice**

^1^Alexander Brückner, ^1^Adrian Brandtner, ^1^Sarah Rieck, ^5^Michaela Matthey, ^1^Caroline Geisen, ^2,3^ Benedikt Fels, ^4^Marta Stei, ^2,3^ Kristina Kusche-Vihrog, ^1^Bernd K Fleischmann, ^1,5^Daniela Wenzel

^1^Institute of Physiology I, Life&Brain Center, Medical Faculty, University of Bonn, Bonn, Germany

^2^Institute of Physiology, University of Lübeck, Lübeck, Germany

^3^DZHK (German Research Centre for Cardiovascular Research), Partner Site Hamburg/Luebeck/Kiel, Luebeck, Germany

^4^Heart Center Bonn, Clinic for Internal Medicine II, University Hospital Bonn, Bonn, Germany

^5^Institute of Physiology, Department of Systems Physiology, Medical Faculty, Ruhr University of Bochum, Bochum, Germany.

*Corresponding author:

Daniela Wenzel, MD

Institute of Physiology, Department of Systems Physiology

Medical Faculty, University of Bochum

Universitätsstr. 150

44801 Bochum

Germany

Tel: 0049/234/32-29100

Email: daniela.wenzel@rub.de

**
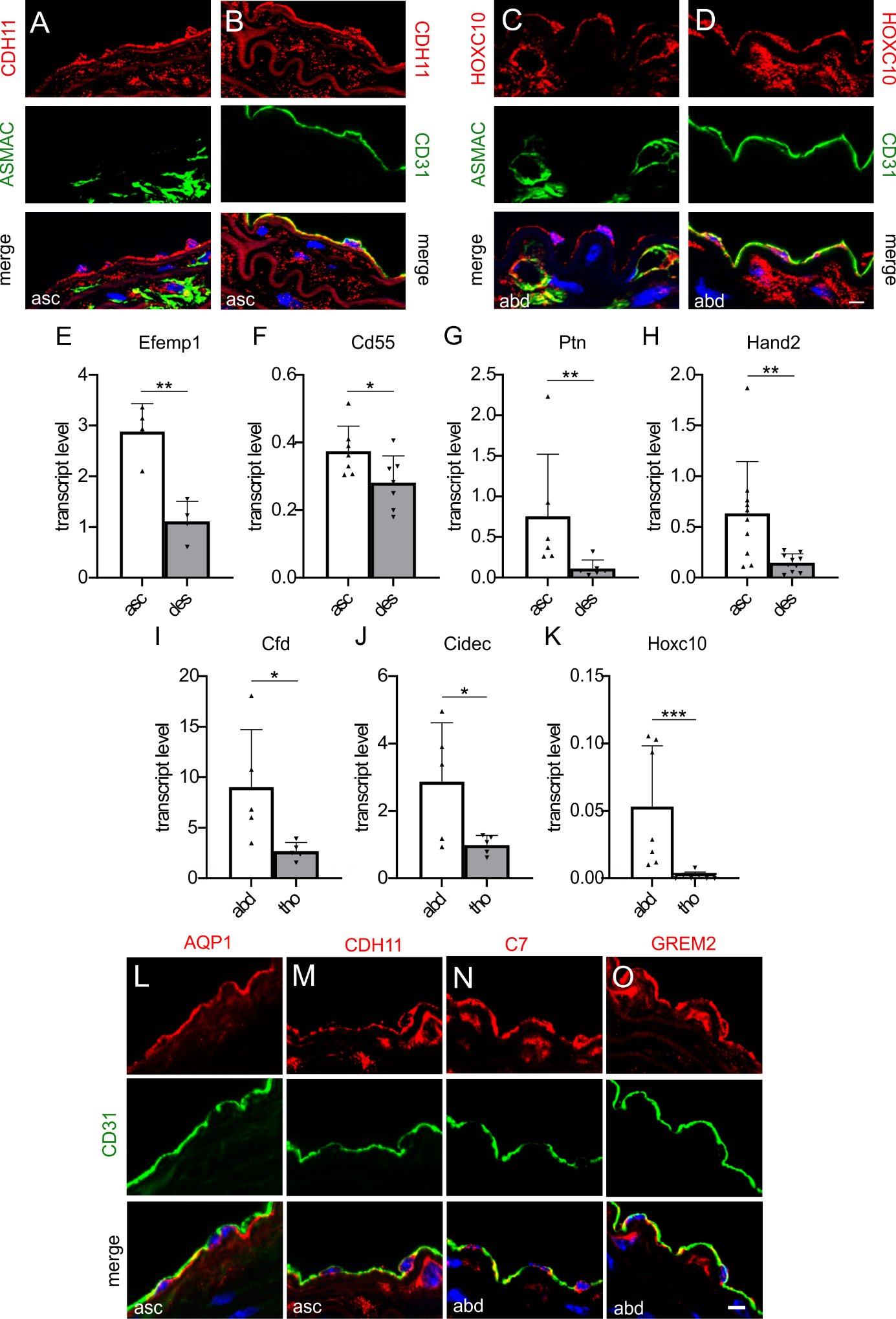
**

**Figure S1. Analysis of EC protein expression by immunofluorescence stainings and mRNA expression by dPCR.**

**A-D)** Analysis of protein expression of CDH11 (A,B) and HOXC10 (C,D) in the ASMAC^+^ smooth muscle layer and CD31^+^ endothelium of aortic segments indicated, scale bar: 5 µm. **E-K)** Comparison of gene expression by dPCR of strongly expressed genes in the endothelium of the ascending (asc) vs descending (des) arch: Efemp1 (E), Cd55 (F), Ptn (G), Hand2 (H) and of the abdominal (abd) vs thoracic (tho) aorta: Cfd (I), Cidec (H) and Hoxc10 (K). Samples for each bar diagram were taken from the same animals. **L-O)** Analysis of protein expression of AQP1 (L), CDH11 (M), C7 (N) and GREM2 (O) in the CD31^+^ endothelium of the aortic segments indicated, scale bar: 5 µm. **E,F,I,J)** unpaired student`s t-test, **G,H,K)** Mann-Whitney U-test, *p<0.05, **p<0.01, ***p<0.001.

**
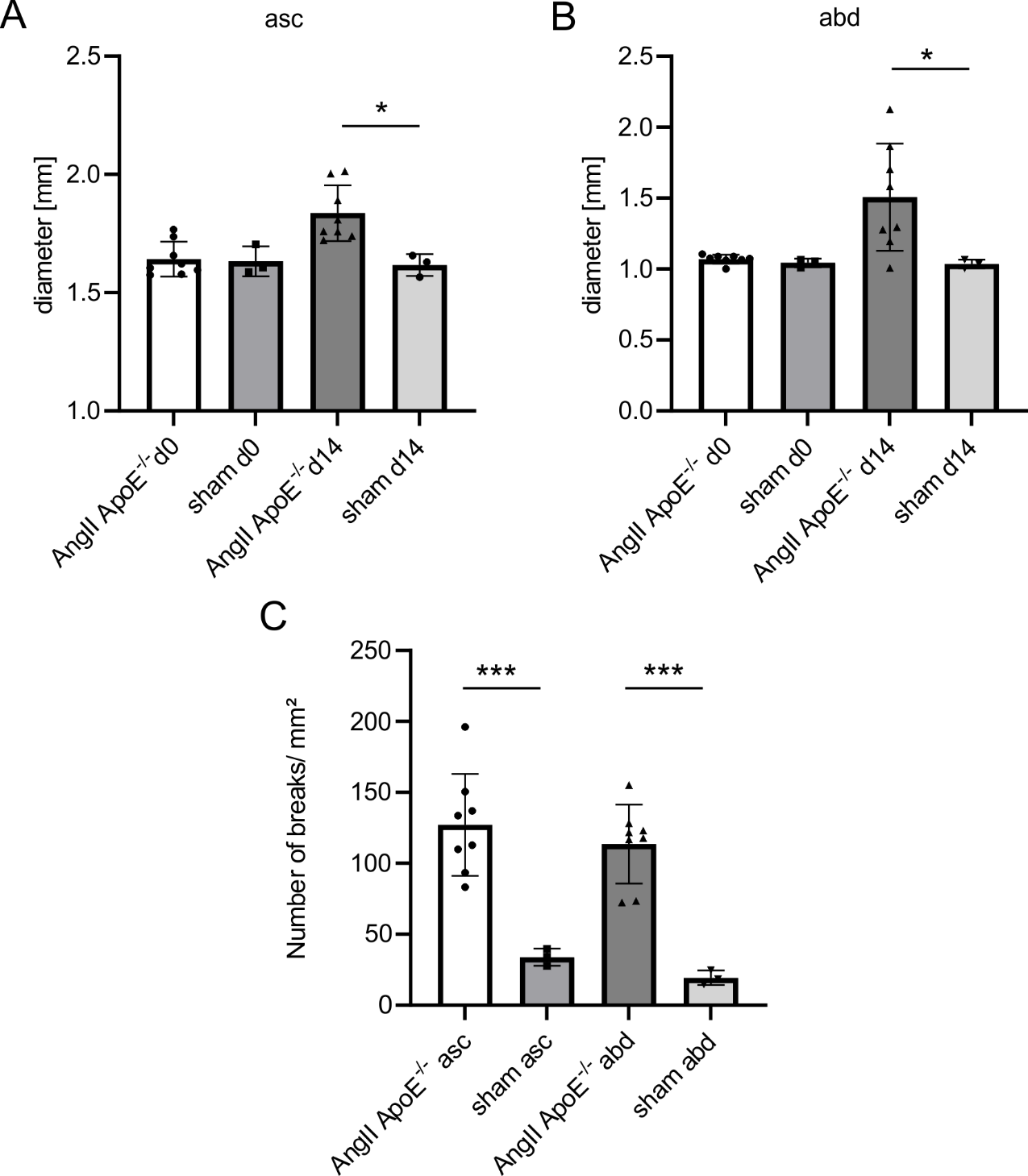
**

**Figure S2. Analysis of aortic diameter and elastin breaks in the ascending arch (asc) and the abdominal aorta (abd).** **A,B)** Statistical analysis of the aortic diameter of the ascending arch (I) and the abdominal aorta (J) in AngII ApoE^-/-^ and sham mice. C**)** Statistical analysis of the number of elastin breaks in the ascending arch and the abdominal aorta at d14. **A,C)** One way ANOVA, Tukey`s post hoc test, **B)** Welch`s ANOVA, Dunnett`s post hoc test, *p<0.05, ***p<0.001.


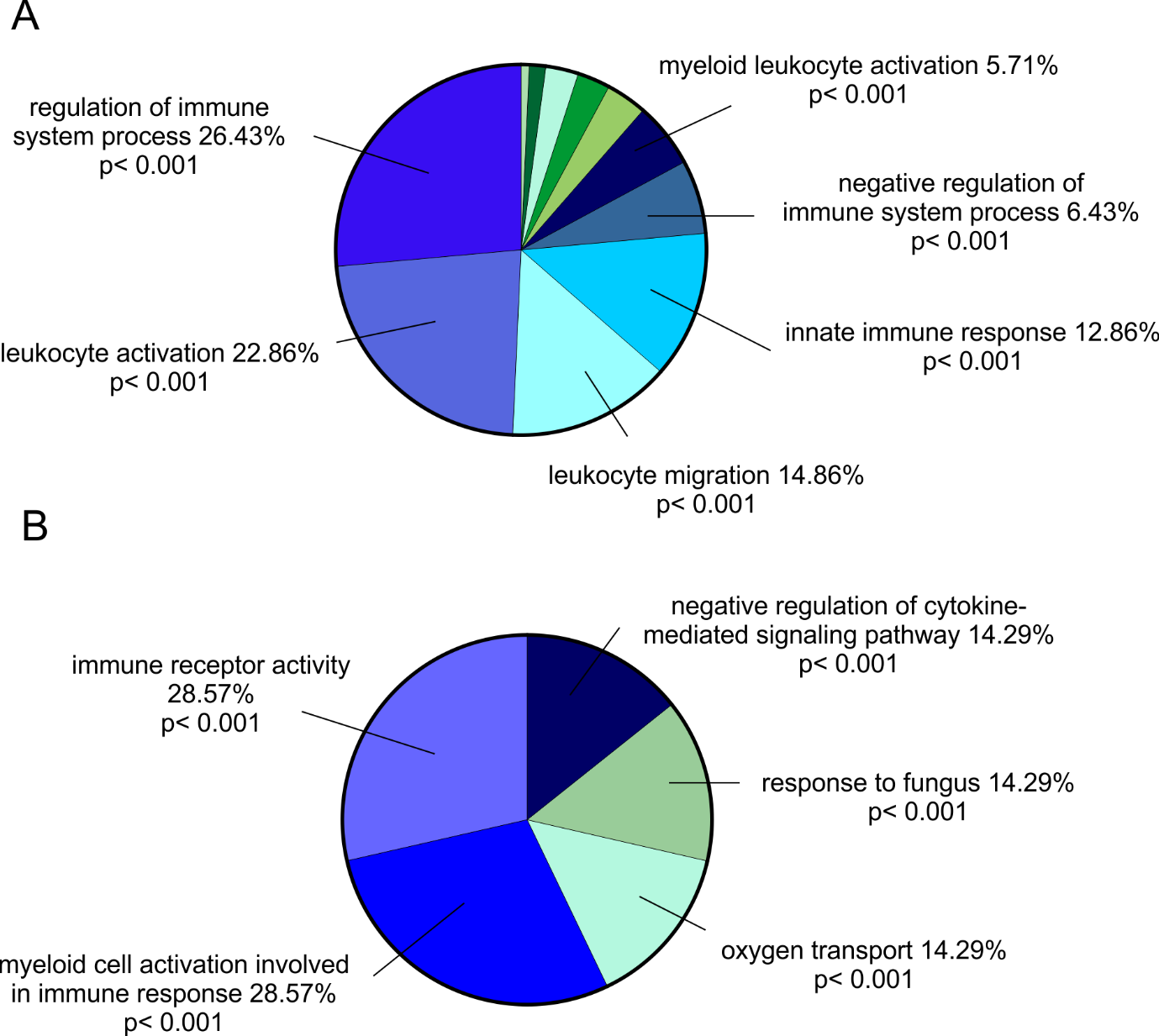


**Figure S3. Differential expression of pro-inflammatory genes in ECs from ApoE^-/-^ mice.**

**A)** GO analysis of the category “biological processes” of the DEGs in ECs from the ascending arch of ApoE^-/-^ mice with western diet vs WT mice. **B)** GO analysis of the category “biological processes” of the DEGs in ECs from the abdominal aorta of ApoE^-/-^ mice with western diet vs WT mice.

**Supplementary Tables**

**Table S1 Differentially expressed genes of asc vs des**

| Genes upregulated in asc | | | |
| --- | --- | --- | --- |
| Symbol | Gen ID | log2(FC) | P adj |
| Gm37580 | ENSMUSG00000102398 | 5.99 | 4.56E-08 |
| Hand2 | ENSMUSG00000038193 | 5.60 | 1.94E-07 |
| Gm45535 | ENSMUSG00000109908 | 5.18 | 1.99E-05 |
| Gm45257 | ENSMUSG00000110084 | 5.00 | 5.78E-05 |
| Epha7 | ENSMUSG00000028289 | 4.97 | 1.11E-05 |
| Diras2 | ENSMUSG00000047842 | 4.79 | 1.32E-04 |
| Samd5 | ENSMUSG00000060487 | 4.62 | 2.80E-06 |
| Fam167a | ENSMUSG00000035095 | 4.36 | 1.12E-03 |
| Cst6 | ENSMUSG00000024846 | 4.08 | 6.52E-03 |
| Ptn | ENSMUSG00000029838 | 4.01 | 5.42E-03 |
| Upk3b | ENSMUSG00000042985 | 3.97 | 3.18E-03 |
| Gm45540 | ENSMUSG00000110397 | 3.83 | 5.09E-03 |
| Cdh11 | ENSMUSG00000031673 | 3.80 | 1.01E-03 |
| Lepr | ENSMUSG00000057722 | 3.75 | 1.12E-03 |
| Sall1 | ENSMUSG00000031665 | 3.71 | 3.18E-03 |
| Mfsd7c | ENSMUSG00000034258 | 3.54 | 0.031 |
| 9430099M06Rik | ENSMUSG00000109997 | 3.54 | 0.044 |
| 2900027M19Rik | ENSMUSG00000109162 | 3.50 | 0.032 |
| Gm22613 | ENSMUSG00000084628 | 3.47 | 0.019 |
| Tbx20 | ENSMUSG00000031965 | 3.46 | 3.22E-04 |
| Gm17910 | ENSMUSG00000110299 | 3.28 | 0.002 |
| H19 | ENSMUSG00000000031 | 3.19 | 0.023 |
| Spry4 | ENSMUSG00000024427 | 3.19 | 0.050 |
| Ccdc141 | ENSMUSG00000044033 | 2.92 | 0.013 |
| Mettl24 | ENSMUSG00000045555 | 2.89 | 0.034 |
| C330027C09Rik | ENSMUSG00000033031 | 2.88 | 0.032 |
| Aldh1a3 | ENSMUSG00000015134 | 2.88 | 0.030 |
| Aqp1 | ENSMUSG00000004655 | 2.82 | 1.20E-04 |
| Ret | ENSMUSG00000030110 | 2.78 | 0.048 |
| Arhgap18 | ENSMUSG00000039031 | 2.35 | 0.014 |
| Top3a | ENSMUSG00000002814 | 2.17 | 0.042 |
| Rab27b | ENSMUSG00000024511 | 2.05 | 0.007 |
| Cd55 | ENSMUSG00000026399 | 1.81 | 0.004 |
| Efemp1 | ENSMUSG00000020467 | 1.81 | 1.40E-06 |
| Olfml2a | ENSMUSG00000046618 | 1.79 | 0.020 |
| Nrbp2 | ENSMUSG00000075590 | 1.52 | 0.023 |
| Stmn2 | ENSMUSG00000027500 | 1.48 | 0.050 |
| Zfand6 | ENSMUSG00000030629 | 1.24 | 0.028 |
| Krcc1 | ENSMUSG00000053012 | 0.78 | 0.014 |

| Genes downregulated in asc | | | |
| --- | --- | --- | --- |
| Symbol | Gen ID | log2(FC) | P adj |
| RP24-538A12.2 | ENSMUSG00000111699 | -4.46 | 7.49E-04 |
| Hoxa7 | ENSMUSG00000038236 | -4.34 | 5.43E-06 |
| Gm20467 | ENSMUSG00000092250 | -4.30 | 1.95E-03 |
| Gm43022 | ENSMUSG00000104547 | -4.22 | 4.04E-07 |
| RP24-126O19.4 | ENSMUSG00000110895 | -3.90 | 6.33E-03 |
| Moxd1 | ENSMUSG00000020000 | -3.69 | 0.020 |
| RP24-89C4.5 | ENSMUSG00000110803 | -3.57 | 0.019 |
| Gm13608 | ENSMUSG00000081480 | -3.53 | 0.037 |
| 6330403L08Rik | ENSMUSG00000075585 | -3.50 | 0.029 |
| Hoxd3os1 | ENSMUSG00000052371 | -3.45 | 0.041 |
| Hoxb9 | ENSMUSG00000020875 | -3.44 | 0.041 |
| Gm16279 | ENSMUSG00000084998 | -3.39 | 0.048 |
| Scd4 | ENSMUSG00000050195 | -3.35 | 0.041 |
| Lrriq3 | ENSMUSG00000028182 | -3.34 | 0.044 |
| Suv39h1 | ENSMUSG00000039231 | -3.12 | 0.013 |
| Cd180 | ENSMUSG00000021624 | -2.92 | 0.042 |
| Gm4876 | ENSMUSG00000054556 | -2.90 | 0.014 |
| Spred3 | ENSMUSG00000037239 | -2.86 | 8.71E-03 |
| Sdr42e1 | ENSMUSG00000034308 | -2.83 | 0.044 |
| Cep170b | ENSMUSG00000072825 | -2.63 | 0.041 |
| Ppcdc | ENSMUSG00000063849 | -2.58 | 0.011 |
| Cdk5rap3 | ENSMUSG00000018669 | -2.55 | 0.011 |
| Cirbp | ENSMUSG00000045193 | -2.51 | 3.65E-03 |
| Gprin3 | ENSMUSG00000045441 | -2.29 | 0.013 |
| Cnp | ENSMUSG00000006782 | -2.23 | 0.017 |
| Hdac9 | ENSMUSG00000004698 | -2.19 | 0.047 |
| Med4 | ENSMUSG00000022109 | -1.84 | 0.013 |
| Dennd4b | ENSMUSG00000042404 | -1.82 | 0.013 |
| Tonsl | ENSMUSG00000059323 | -1.60 | 0.050 |

**Table S2 Differentially expressed genes of asc vs tho**

| Genes upregulated in asc | | | |
| --- | --- | --- | --- |
| Symbol | Gen ID | log2(FC) | P adj |
| Ptn | ENSMUSG00000029838 | 8.09 | 8.25E-20 |
| Abcb1a | ENSMUSG00000040584 | 7.07 | 3.12E-15 |
| Sez6l | ENSMUSG00000058153 | 6.28 | 1.32E-09 |
| 5033428I22Rik | ENSMUSG00000097910 | 6.21 | 7.55E-10 |
| Hand2 | ENSMUSG00000038193 | 5.82 | 4.60E-08 |
| Gm45540 | ENSMUSG00000110397 | 5.64 | 7.14E-07 |
| Dcdc2a | ENSMUSG00000035910 | 5.55 | 1.70E-08 |
| Gm45535 | ENSMUSG00000109908 | 5.48 | 6.72E-06 |
| Six2 | ENSMUSG00000024134 | 5.36 | 5.03E-09 |
| Gm45257 | ENSMUSG00000110084 | 5.32 | 1.64E-05 |
| Sall1 | ENSMUSG00000031665 | 5.32 | 5.25E-06 |
| Mmp12 | ENSMUSG00000049723 | 5.31 | 4.63E-09 |
| Ccr5 | ENSMUSG00000079227 | 5.26 | 4.17E-07 |
| Fam167a | ENSMUSG00000035095 | 5.20 | 2.07E-05 |
| Trpc6 | ENSMUSG00000031997 | 5.18 | 2.68E-05 |
| Gm35339 | ENSMUSG00000109179 | 5.17 | 2.33E-08 |
| Mettl24 | ENSMUSG00000045555 | 5.13 | 7.86E-06 |
| Slamf7 | ENSMUSG00000038179 | 5.07 | 2.23E-06 |
| Ttc9 | ENSMUSG00000042734 | 5.00 | 1.40E-05 |
| Diras2 | ENSMUSG00000047842 | 4.81 | 1.33E-04 |
| Samd5 | ENSMUSG00000060487 | 4.67 | 5.19E-05 |
| Nckap5 | ENSMUSG00000049690 | 4.53 | 9.16E-04 |
| Stra6 | ENSMUSG00000032327 | 4.49 | 5.65E-04 |
| Ms4a7 | ENSMUSG00000024672 | 4.48 | 1.32E-03 |
| Ormdl1 | ENSMUSG00000026097 | 4.47 | 7.61E-04 |
| Chn2 | ENSMUSG00000004633 | 4.46 | 2.29E-18 |
| Lmnb2 | ENSMUSG00000062075 | 4.46 | 5.56E-04 |
| Padi2 | ENSMUSG00000028927 | 4.43 | 2.04E-04 |
| Cst6 | ENSMUSG00000024846 | 4.42 | 1.02E-03 |
| Trim9 | ENSMUSG00000021071 | 4.39 | 3.08E-04 |
| Gm43557 | ENSMUSG00000105212 | 4.39 | 9.28E-04 |
| St8sia2 | ENSMUSG00000025789 | 4.38 | 4.51E-05 |
| Gmnn | ENSMUSG00000006715 | 4.37 | 4.43E-05 |
| Slamf1 | ENSMUSG00000015316 | 4.37 | 1.04E-03 |
| Cdh11 | ENSMUSG00000031673 | 4.36 | 1.78E-06 |
| Ret | ENSMUSG00000030110 | 4.26 | 2.23E-05 |
| Pcdh1 | ENSMUSG00000051375 | 4.19 | 5.09E-03 |
| Cxcl2 | ENSMUSG00000058427 | 4.13 | 3.81E-03 |
| Gm45263 | ENSMUSG00000110342 | 4.11 | 3.64E-03 |
| Pcdh12 | ENSMUSG00000024440 | 4.07 | 4.28E-03 |
| Epha7 | ENSMUSG00000028289 | 4.07 | 5.09E-03 |
| AV026068 | ENSMUSG00000100510 | 4.05 | 4.37E-03 |
| Dkk2 | ENSMUSG00000028031 | 4.01 | 2.46E-04 |
| Tuft1 | ENSMUSG00000005968 | 4.01 | 0.010 |
| Gm15267 | ENSMUSG00000083474 | 4.00 | 4.28E-03 |
| Gm42981 | ENSMUSG00000105282 | 3.98 | 1.53E-04 |
| P2ry14 | ENSMUSG00000036381 | 3.97 | 7.90E-03 |
| RP23-440L7.5 | ENSMUSG00000111160 | 3.93 | 2.10E-03 |
| Nostrin | ENSMUSG00000034738 | 3.88 | 4.28E-03 |
| Rabl3 | ENSMUSG00000022827 | 3.87 | 7.77E-04 |
| Itgax | ENSMUSG00000030789 | 3.86 | 3.75E-03 |
| BC028528 | ENSMUSG00000038543 | 3.85 | 5.39E-03 |
| Gm42982 | ENSMUSG00000105503 | 3.82 | 0.019 |
| Far1os | ENSMUSG00000084984 | 3.77 | 7.77E-04 |
| 9430099M06Rik | ENSMUSG00000109997 | 3.76 | 0.024 |
| Arhgap27 | ENSMUSG00000034255 | 3.76 | 0.019 |
| Hecw2 | ENSMUSG00000042807 | 3.71 | 0.022 |
| Klra2 | ENSMUSG00000030187 | 3.64 | 0.030 |
| 2900027M19Rik | ENSMUSG00000109162 | 3.64 | 0.019 |
| Ly9 | ENSMUSG00000004707 | 3.62 | 0.033 |
| 4933400A11Rik | ENSMUSG00000055357 | 3.61 | 0.030 |
| A4galt | ENSMUSG00000047878 | 3.60 | 4.91E-04 |
| Rundc3b | ENSMUSG00000040570 | 3.59 | 0.029 |
| Gm20756 | ENSMUSG00000106799 | 3.58 | 0.025 |
| Dnase1l3 | ENSMUSG00000025279 | 3.56 | 8.81E-03 |
| Gm44175 | ENSMUSG00000108132 | 3.56 | 0.036 |
| Dync1i1 | ENSMUSG00000029757 | 3.54 | 0.041 |
| Gpr65 | ENSMUSG00000021886 | 3.53 | 0.015 |
| Slc7a8 | ENSMUSG00000022180 | 3.52 | 0.019 |
| Rem1 | ENSMUSG00000000359 | 3.50 | 0.041 |
| Astn2 | ENSMUSG00000028373 | 3.48 | 0.035 |
| Sdk2 | ENSMUSG00000041592 | 3.48 | 0.031 |
| Cry1 | ENSMUSG00000020038 | 3.47 | 4.37E-03 |
| Msantd3 | ENSMUSG00000039693 | 3.44 | 0.029 |
| Mief2 | ENSMUSG00000018599 | 3.41 | 0.041 |
| Mettl15 | ENSMUSG00000057234 | 3.36 | 0.022 |
| 2010109A12Rik | ENSMUSG00000029384 | 3.34 | 0.036 |
| Ccdc39 | ENSMUSG00000027676 | 3.33 | 3.44E-04 |
| 4921513I03Rik | ENSMUSG00000044544 | 3.23 | 0.019 |
| Tmem45a | ENSMUSG00000022754 | 3.22 | 4.37E-03 |
| Tmem106a | ENSMUSG00000034947 | 3.20 | 2.93E-04 |
| 3000002C10Rik | ENSMUSG00000070282 | 3.16 | 0.030 |
| Gm37745 | ENSMUSG00000103146 | 3.10 | 0.016 |
| Opcml | ENSMUSG00000062257 | 3.04 | 0.048 |
| Pcdhb19 | ENSMUSG00000043313 | 3.00 | 0.038 |
| Aqp1 | ENSMUSG00000004655 | 2.98 | 0.018 |
| Fam134b | ENSMUSG00000022270 | 2.95 | 0.015 |
| Pthlh | ENSMUSG00000048776 | 2.95 | 4.44E-03 |
| Ccdc141 | ENSMUSG00000044033 | 2.95 | 0.021 |
| Fxyd6 | ENSMUSG00000066705 | 2.93 | 0.034 |
| Ifitm10 | ENSMUSG00000045777 | 2.79 | 0.045 |
| Junos | ENSMUSG00000087366 | 2.77 | 0.043 |
| Cbr3 | ENSMUSG00000022947 | 2.65 | 0.015 |
| Chn1 | ENSMUSG00000056486 | 2.51 | 0.033 |
| Dlx6os1 | ENSMUSG00000090063 | 2.48 | 0.028 |
| Mettl8 | ENSMUSG00000041975 | 2.43 | 0.037 |
| C1galt1c1 | ENSMUSG00000048970 | 2.36 | 0.038 |
| Fgl2 | ENSMUSG00000039899 | 2.27 | 7.41E-05 |
| Cbwd1 | ENSMUSG00000024878 | 2.17 | 0.032 |
| Rab27b | ENSMUSG00000024511 | 2.12 | 1.10E-03 |
| Cd74 | ENSMUSG00000024610 | 2.11 | 5.28E-04 |
| Serpina1d | ENSMUSG00000071177 | 2.09 | 1.91E-03 |
| Sema3d | ENSMUSG00000040254 | 2.04 | 4.28E-03 |
| Laptm5 | ENSMUSG00000028581 | 2.02 | 0.037 |
| Efemp1 | ENSMUSG00000020467 | 1.96 | 6.00E-06 |
| Pald1 | ENSMUSG00000020092 | 1.82 | 7.77E-04 |
| Cdh13 | ENSMUSG00000031841 | 1.71 | 1.09E-04 |
| Zbtb25 | ENSMUSG00000056459 | 1.70 | 0.037 |
| Anpep | ENSMUSG00000039062 | 1.65 | 0.015 |
| Snx6 | ENSMUSG00000005656 | 1.54 | 0.036 |
| 2010111I01Rik | ENSMUSG00000021458 | 1.33 | 0.015 |
| Cd9 | ENSMUSG00000030342 | 1.33 | 3.62E-04 |
| Fry | ENSMUSG00000056602 | 1.30 | 0.015 |

| Genes downregulated in asc | | | |
| --- | --- | --- | --- |
| Symbol | Gen ID | log2(FC) | P adj |
| Hoxb9 | ENSMUSG00000020875 | -7.18 | 6.97E-19 |
| Hoxd3os1 | ENSMUSG00000052371 | -5.74 | 2.06E-09 |
| Hoxb7 | ENSMUSG00000038721 | -5.71 | 5.03E-08 |
| Slc26a4 | ENSMUSG00000020651 | -5.64 | 6.01E-10 |
| Hoxc8 | ENSMUSG00000001657 | -5.54 | 1.33E-06 |
| Hoxa7 | ENSMUSG00000038236 | -5.40 | 8.56E-11 |
| Gm53 | ENSMUSG00000078706 | -5.13 | 2.92E-05 |
| Hoxb6 | ENSMUSG00000000690 | -4.88 | 2.68E-05 |
| Hoxb5 | ENSMUSG00000038700 | -4.59 | 2.79E-05 |
| RP24-538A12.2 | ENSMUSG00000111699 | -4.39 | 1.32E-03 |
| Hoxb8 | ENSMUSG00000056648 | -4.31 | 1.68E-03 |
| Gm16126 | ENSMUSG00000086585 | -4.02 | 5.89E-03 |
| Gm36266 | ENSMUSG00000106617 | -3.76 | 0.012 |
| Fosb | ENSMUSG00000003545 | -3.72 | 0.018 |
| Kcnq4 | ENSMUSG00000028631 | -3.64 | 4.60E-05 |
| Nek3 | ENSMUSG00000031478 | -3.50 | 0.016 |
| Hoxa9 | ENSMUSG00000038227 | -3.47 | 2.12E-04 |
| RP24-126O19.4 | ENSMUSG00000110895 | -3.46 | 0.042 |
| Gm26632 | ENSMUSG00000097442 | -3.39 | 0.012 |
| Gprin3 | ENSMUSG00000045441 | -3.10 | 1.14E-14 |
| Cdpf1 | ENSMUSG00000064284 | -3.03 | 0.049 |
| 0610009B22Rik | ENSMUSG00000007777 | -2.89 | 2.51E-03 |
| Rrp36 | ENSMUSG00000023971 | -2.81 | 0.029 |
| Gatsl3 | ENSMUSG00000020424 | -2.69 | 0.043 |
| Adra2a | ENSMUSG00000033717 | -2.61 | 0.035 |
| Spred3 | ENSMUSG00000037239 | -2.51 | 0.038 |
| Pelp1 | ENSMUSG00000018921 | -2.25 | 0.014 |
| Adh7 | ENSMUSG00000055301 | -2.19 | 2.93E-04 |
| Pcdh9 | ENSMUSG00000055421 | -1.95 | 0.015 |
| Med4 | ENSMUSG00000022109 | -1.91 | 0.031 |
| Pitpnm3 | ENSMUSG00000040543 | -1.83 | 0.030 |
| Lrrc32 | ENSMUSG00000090958 | -1.61 | 1.99E-07 |
| Kdm6b | ENSMUSG00000018476 | -1.34 | 0.019 |
| Gprasp1 | ENSMUSG00000043384 | -1.26 | 0.013 |
| Sik3 | ENSMUSG00000034135 | -1.22 | 2.46E-04 |
| Uaca | ENSMUSG00000034485 | -1.19 | 8.45E-03 |
| Twist1 | ENSMUSG00000035799 | -1.19 | 0.026 |
| Ptch1 | ENSMUSG00000021466 | -1.15 | 0.015 |
| Dcp2 | ENSMUSG00000024472 | -1.06 | 5.09E-03 |
| Ehd4 | ENSMUSG00000027293 | -0.99 | 6.84E-03 |
| Ablim1 | ENSMUSG00000025085 | -0.81 | 0.034 |

**Table S3 Differentially expressed genes of asc vs abd**

| Genes upregulated in asc | | | |
| --- | --- | --- | --- |
| Symbol | Gen ID | log2(FC) | P adj |
| Tbx20 | ENSMUSG00000031965 | 8.17 | 4.45E-10 |
| Cdh11 | ENSMUSG00000031673 | 6.87 | 9.09E-10 |
| 5033428I22Rik | ENSMUSG00000097910 | 6.59 | 1.01E-05 |
| Tifab | ENSMUSG00000049625 | 6.07 | 1.53E-06 |
| Dsg2 | ENSMUSG00000044393 | 6.05 | 2.44E-07 |
| Dcdc2a | ENSMUSG00000035910 | 6.02 | 5.08E-06 |
| Sez6l | ENSMUSG00000058153 | 5.99 | 2.14E-05 |
| Gm45535 | ENSMUSG00000109908 | 5.95 | 9.16E-04 |
| Fam167a | ENSMUSG00000035095 | 5.90 | 2.05E-03 |
| Sox8 | ENSMUSG00000024176 | 5.90 | 1.07E-03 |
| Tcf23 | ENSMUSG00000006642 | 5.83 | 2.27E-06 |
| Gm19967 | ENSMUSG00000093858 | 5.82 | 3.46E-06 |
| Cd300ld | ENSMUSG00000034641 | 5.79 | 6.98E-06 |
| Sfmbt2 | ENSMUSG00000061186 | 5.70 | 2.95E-06 |
| Gm45257 | ENSMUSG00000110084 | 5.65 | 1.75E-03 |
| Sdk2 | ENSMUSG00000041592 | 5.53 | 1.04E-05 |
| Sdk1 | ENSMUSG00000039683 | 5.52 | 1.87E-03 |
| Gm45512 | ENSMUSG00000110071 | 5.52 | 2.30E-04 |
| Lrrn1 | ENSMUSG00000034648 | 5.49 | 1.86E-03 |
| Wnt9b | ENSMUSG00000018486 | 5.49 | 0.012 |
| AV026068 | ENSMUSG00000100510 | 5.46 | 5.09E-03 |
| Kcnrg | ENSMUSG00000046168 | 5.44 | 6.98E-06 |
| Ccnb2-ps | ENSMUSG00000083718 | 5.41 | 1.93E-05 |
| Gm16192 | ENSMUSG00000086773 | 5.38 | 2.65E-05 |
| Dkk2 | ENSMUSG00000028031 | 5.37 | 2.30E-04 |
| C330027C09Rik | ENSMUSG00000033031 | 5.35 | 5.36E-04 |
| Hand2 | ENSMUSG00000038193 | 5.33 | 1.25E-04 |
| Adgrl3 | ENSMUSG00000037605 | 5.33 | 2.60E-04 |
| A430110L20Rik | ENSMUSG00000054135 | 5.26 | 3.56E-05 |
| Mal | ENSMUSG00000027375 | 5.22 | 1.21E-06 |
| Trpc6 | ENSMUSG00000031997 | 5.18 | 4.36E-03 |
| Serpina1b | ENSMUSG00000071178 | 5.16 | 2.48E-05 |
| A130014A01Rik | ENSMUSG00000097944 | 5.15 | 2.60E-04 |
| Gm37788 | ENSMUSG00000102326 | 5.13 | 8.70E-04 |
| Gm37109 | ENSMUSG00000102139 | 5.13 | 0.014 |
| Sall1 | ENSMUSG00000031665 | 5.08 | 2.22E-03 |
| Gata4 | ENSMUSG00000021944 | 5.04 | 0.012 |
| Gal3st3 | ENSMUSG00000047658 | 4.90 | 0.012 |
| Gm45632 | ENSMUSG00000110077 | 4.90 | 0.011 |
| 4930517O19Rik | ENSMUSG00000054391 | 4.86 | 1.80E-03 |
| Hmmr | ENSMUSG00000020330 | 4.86 | 4.44E-04 |
| 2810428J06Rik | ENSMUSG00000105981 | 4.85 | 7.22E-05 |
| Gm15563 | ENSMUSG00000086387 | 4.83 | 0.012 |
| Gm15951 | ENSMUSG00000081301 | 4.75 | 0.029 |
| Mmp12 | ENSMUSG00000049723 | 4.74 | 2.56E-03 |
| Efna1 | ENSMUSG00000027954 | 4.72 | 9.16E-04 |
| C030034I22Rik | ENSMUSG00000073374 | 4.71 | 0.0191 |
| Mettl24 | ENSMUSG00000045555 | 4.71 | 3.78E-03 |
| Hapln1 | ENSMUSG00000021613 | 4.70 | 0.049 |
| Gm31049 | ENSMUSG00000106996 | 4.69 | 0.037 |
| Gm37580 | ENSMUSG00000102398 | 4.67 | 0.011 |
| Gm17910 | ENSMUSG00000110299 | 4.62 | 0.013 |
| Cxxc4 | ENSMUSG00000044365 | 4.60 | 0.036 |
| B430203G13Rik | ENSMUSG00000067356 | 4.58 | 0.044 |
| H2-M2 | ENSMUSG00000016283 | 4.57 | 0.049 |
| Rem1 | ENSMUSG00000000359 | 4.56 | 0.043 |
| Cubn | ENSMUSG00000026726 | 4.52 | 8.45E-03 |
| Mmp13 | ENSMUSG00000050578 | 4.51 | 0.049 |
| Ciart | ENSMUSG00000038550 | 4.47 | 1.60E-03 |
| Marveld2 | ENSMUSG00000021636 | 4.45 | 0.021 |
| Gm15915 | ENSMUSG00000085723 | 4.45 | 0.043 |
| Gm15267 | ENSMUSG00000083474 | 4.43 | 0.044 |
| Cst6 | ENSMUSG00000024846 | 4.41 | 0.033 |
| Tnnt2 | ENSMUSG00000026414 | 4.37 | 0.017 |
| Ttc9 | ENSMUSG00000042734 | 4.37 | 0.011 |
| Rsf1os2 | ENSMUSG00000086993 | 4.34 | 0.049 |
| Zfp185 | ENSMUSG00000031351 | 4.33 | 0.019 |
| Gmnn | ENSMUSG00000006715 | 4.32 | 0.021 |
| Scml4 | ENSMUSG00000044770 | 4.31 | 0.015 |
| Amelx | ENSMUSG00000031354 | 4.31 | 3.45E-03 |
| Astn2 | ENSMUSG00000028373 | 4.30 | 0.049 |
| Gm42981 | ENSMUSG00000105282 | 4.30 | 4.74E-03 |
| Pomgnt2 | ENSMUSG00000066235 | 4.29 | 0.045 |
| B230344G16Rik | ENSMUSG00000097800 | 4.28 | 0.044 |
| Gm20756 | ENSMUSG00000106799 | 4.23 | 0.049 |
| Best1 | ENSMUSG00000037418 | 4.22 | 8.96E-03 |
| Kazn | ENSMUSG00000040606 | 4.21 | 0.034 |
| Ppp1r13l | ENSMUSG00000040734 | 4.19 | 0.049 |
| Dlg2 | ENSMUSG00000052572 | 4.18 | 0.021 |
| 2510016D11Rik | ENSMUSG00000106825 | 4.17 | 0.018 |
| Slco1b2 | ENSMUSG00000030236 | 4.16 | 0.049 |
| Gm37238 | ENSMUSG00000103373 | 4.16 | 4.74E-03 |
| Gm43793 | ENSMUSG00000107155 | 4.15 | 4.44E-04 |
| Dkk3 | ENSMUSG00000030772 | 4.15 | 5.60E-03 |
| Dlx6os1 | ENSMUSG00000090063 | 4.12 | 5.09E-03 |
| Ppp1r16a | ENSMUSG00000033819 | 4.12 | 0.050 |
| Gm37320 | ENSMUSG00000104324 | 4.10 | 9.16E-04 |
| Trim9 | ENSMUSG00000021071 | 4.09 | 0.034 |
| 9130023H24Rik | ENSMUSG00000062944 | 4.09 | 0.012 |
| Gm15859 | ENSMUSG00000086944 | 4.09 | 0.049 |
| 2610001A08Rik | ENSMUSG00000103828 | 4.08 | 0.036 |
| Gm44432 | ENSMUSG00000107932 | 4.08 | 0.047 |
| Lat | ENSMUSG00000030742 | 4.06 | 0.016 |
| Gm38042 | ENSMUSG00000104292 | 4.06 | 0.024 |
| Gm12743 | ENSMUSG00000084968 | 4.05 | 0.024 |
| Gm44777 | ENSMUSG00000108738 | 4.03 | 0.021 |
| Ret | ENSMUSG00000030110 | 4.03 | 5.36E-04 |
| Gm16068 | ENSMUSG00000086121 | 3.97 | 0.049 |
| 4930405O22Rik | ENSMUSG00000090565 | 6.96 | 0.049 |
| Gm19557 | ENSMUSG00000097990 | 3.93 | 0.020 |
| Smpdl3b | ENSMUSG00000028885 | 3.89 | 0.049 |
| Dnase1l3 | ENSMUSG00000025279 | 3.88 | 0.038 |
| Ifitm10 | ENSMUSG00000045777 | 3.86 | 0.049 |
| Ccdc39 | ENSMUSG00000027676 | 3.84 | 0.049 |
| Rbm4 | ENSMUSG00000094936 | 3.80 | 0.012 |
| Fbxl7 | ENSMUSG00000043556 | 3.73 | 0.038 |
| 3000002C10Rik | ENSMUSG00000070282 | 3.72 | 0.031 |
| Gm43679 | ENSMUSG00000105950 | 3.68 | 0.049 |
| Cthrc1 | ENSMUSG00000054196 | 3.68 | 0.017 |
| Trim13 | ENSMUSG00000035235 | 3.68 | 0.019 |
| 4933407K13Rik | ENSMUSG00000087396 | 3.65 | 0.049 |
| Mmp16 | ENSMUSG00000028226 | 3.64 | 0.016 |
| Zcchc3 | ENSMUSG00000074682 | 3.60 | 0.049 |
| Zbtb7c | ENSMUSG00000044646 | 3.60 | 0.024 |
| Ccdc68 | ENSMUSG00000038903 | 3.55 | 0.034 |
| Igsf9 | ENSMUSG00000037995 | 3.53 | 0.049 |
| Fam78b | ENSMUSG00000060568 | 3.53 | 0.043 |
| Cbr3 | ENSMUSG00000022947 | 3.52 | 0.039 |
| Gm12961 | ENSMUSG00000082686 | 3.52 | 0.034 |
| Serpina1d | ENSMUSG00000071177 | 3.43 | 0.027 |
| Gm27010 | ENSMUSG00000098183 | 3.43 | 0.036 |
| Samd14 | ENSMUSG00000047181 | 3.42 | 0.043 |
| Gm1976 | ENSMUSG00000066057 | 3.38 | 0.049 |
| Camsap3 | ENSMUSG00000044433 | 3.37 | 5.31E-03 |
| Ino80dos | ENSMUSG00000084799 | 3.33 | 0.043 |
| Eva1c | ENSMUSG00000039903 | 3.25 | 0.017 |
| Ctc1 | ENSMUSG00000020898 | 3.22 | 0.043 |
| Gm44686 | ENSMUSG00000108563 | 3.19 | 1.75E-03 |
| Tle2 | ENSMUSG00000034771 | 3.17 | 0.025 |
| Hpse | ENSMUSG00000035273 | 3.13 | 0.034 |
| Aqp1 | ENSMUSG00000004655 | 3.10 | 1.01E-03 |
| Sec23b | ENSMUSG00000027429 | 3.08 | 0.036 |
| E4f1 | ENSMUSG00000024137 | 3.03 | 0.044 |
| AI467606 | ENSMUSG00000045165 | 3.03 | 0.049 |
| Lmf2 | ENSMUSG00000022614 | 2.96 | 0.049 |
| Cyp2d22 | ENSMUSG00000061740 | 2.96 | 0.049 |
| Tarbp1 | ENSMUSG00000090290 | 2.93 | 0.049 |
| Igsf3 | ENSMUSG00000042035 | 2.88 | 0.042 |
| C1qa | ENSMUSG00000036887 | 2.87 | 0.033 |
| Olfml2a | ENSMUSG00000046618 | 2.86 | 0.027 |
| Pigl | ENSMUSG00000014245 | 2.75 | 0.045 |
| Chic1 | ENSMUSG00000031327 | 2.71 | 0.044 |
| Serpini1 | ENSMUSG00000027834 | 2.61 | 0.049 |
| Gm42576 | ENSMUSG00000107205 | 2.58 | 0.028 |
| 4632427E13Rik | ENSMUSG00000074024 | 2.53 | 0.035 |
| Ncam1 | ENSMUSG00000039542 | 2.34 | 1.93E-05 |
| Pcdhgc3 | ENSMUSG00000102918 | 2.31 | 0.042 |
| Mgat5 | ENSMUSG00000036155 | 2.18 | 0.035 |
| Car8 | ENSMUSG00000041261 | 2.12 | 0.027 |
| Rab27b | ENSMUSG00000024511 | 2.07 | 0.049 |
| Fgl2 | ENSMUSG00000039899 | 1.79 | 0.036 |
| Fam198b | ENSMUSG00000027955 | 1.70 | 0.021 |
| Fndc1 | ENSMUSG00000071984 | 1.64 | 0.015 |
| Cd9 | ENSMUSG00000030342 | 1.48 | 0.044 |
| Tmem2 | ENSMUSG00000024754 | 1.44 | 0.049 |

| Genes downregulated in asc | | | |
| --- | --- | --- | --- |
| Symbol | Gen ID | log2(FC) | P adj |
| Hoxc8 | ENSMUSG00000001657 | -8.26 | 9.21E-09 |
| Hoxc10 | ENSMUSG00000022484 | -7.72 | 2.46E-06 |
| Gm53 | ENSMUSG00000078706 | -7.32 | 2.46E-06 |
| Hoxb7 | ENSMUSG00000038721 | -6.99 | 1.10E-05 |
| Hoxb9 | ENSMUSG00000020875 | -6.33 | 1.30E-04 |
| Hoxb8 | ENSMUSG00000056648 | -6.21 | 6.76E-05 |
| Hoxd3os1 | ENSMUSG00000052371 | -5.64 | 1.10E-04 |
| Klrk1 | ENSMUSG00000030149 | -5.44 | 0.014 |
| Hoxb6 | ENSMUSG00000000690 | -5.26 | 5.57E-04 |
| Hoxa7 | ENSMUSG00000038236 | -5.20 | 1.75E-03 |
| Gm11837 | ENSMUSG00000086587 | -5.07 | 0.029 |
| Cntfr | ENSMUSG00000028444 | -4.89 | 0.013 |
| Hoxc5 | ENSMUSG00000022485 | -4.86 | 0.049 |
| Inmt | ENSMUSG00000003477 | -4.79 | 1.01E-03 |
| Gm45244 | ENSMUSG00000109807 | -4.68 | 0.044 |
| Snx20 | ENSMUSG00000031662 | -4.63 | 0.012 |
| Epha5 | ENSMUSG00000029245 | -4.60 | 0.049 |
| Mvd | ENSMUSG00000006517 | -4.53 | 0.049 |
| 0610009B22Rik | ENSMUSG00000007777 | -4.42 | 2.44E-03 |
| Spout1 | ENSMUSG00000039660 | -4.33 | 0.024 |
| Rbm20 | ENSMUSG00000043639 | -3.99 | 0.021 |
| Cacnb1 | ENSMUSG00000020882 | -3.95 | 0.049 |
| Dnah10 | ENSMUSG00000038011 | -3.95 | 0.039 |
| Ccl27a | ENSMUSG00000073888 | -3.90 | 0.020 |
| Sdr42e1 | ENSMUSG00000034308 | -3.82 | 0.049 |
| Ablim2 | ENSMUSG00000029095 | -3.79 | 0.028 |
| Srpx | ENSMUSG00000090084 | -3.69 | 0.042 |
| Retn | ENSMUSG00000012705 | -3.67 | 0.022 |
| Paox | ENSMUSG00000025464 | -3.63 | 0.049 |
| Myom1 | ENSMUSG00000024049 | -3.61 | 0.034 |
| Orm1 | ENSMUSG00000039196 | -3.58 | 0.049 |
| Cirbp | ENSMUSG00000045193 | -3.52 | 0.038 |
| Synm | ENSMUSG00000030554 | -3.52 | 0.027 |
| Snta1 | ENSMUSG00000027488 | -3.52 | 0.019 |
| Gys1 | ENSMUSG00000003865 | -3.51 | 0.014 |
| Hoxa9 | ENSMUSG00000038227 | -3.43 | 0.049 |
| Gria4 | ENSMUSG00000025892 | -3.42 | 0.026 |
| Ctcflos | ENSMUSG00000087382 | -3.38 | 0.039 |
| C330018D20Rik | ENSMUSG00000024592 | -3.36 | 0.012 |
| Tpi1 | ENSMUSG00000023456 | -3.36 | 0.028 |
| Prrx1 | ENSMUSG00000026586 | -3.33 | 0.012 |
| Bank1 | ENSMUSG00000037922 | -3.29 | 0.049 |
| Irak1bp1 | ENSMUSG00000032251 | -3.23 | 0.049 |
| Cdc34 | ENSMUSG00000020307 | -3.15 | 0.049 |
| Cyb5d2 | ENSMUSG00000057778 | -3.12 | 0.049 |
| Kbtbd11 | ENSMUSG00000055675 | -3.10 | 3.33E-03 |
| Gm1966 | ENSMUSG00000073902 | -3.01 | 0.033 |
| Sephs2 | ENSMUSG00000049091 | -2.84 | 0.049 |
| Snhg8 | ENSMUSG00000104960 | -2.77 | 0.033 |
| Drap1 | ENSMUSG00000024914 | -2.75 | 0.025 |
| Epb41l1 | ENSMUSG00000027624 | -2.54 | 0.020 |
| Cnp | ENSMUSG00000006782 | -2.45 | 0.014 |
| Gprin3 | ENSMUSG00000045441 | -2.40 | 0.027 |
| Acat2 | ENSMUSG00000023832 | -2.39 | 0.012 |
| Prkrir | ENSMUSG00000030753 | -2.37 | 0.049 |
| Ifi207 | ENSMUSG00000073490 | -2.37 | 0.049 |
| Cacfd1 | ENSMUSG00000015488 | -2.34 | 0.049 |
| Mrpl9 | ENSMUSG00000028140 | -2.31 | 0.049 |
| Irs1 | ENSMUSG00000055980 | -2.27 | 0.049 |
| Selenow | ENSMUSG00000041571 | -2.26 | 0.049 |
| Tonsl | ENSMUSG00000059323 | -2.12 | 0.029 |
| Uqcrb | ENSMUSG00000021520 | -2.05 | 0.044 |
| Adamtsl5 | ENSMUSG00000043822 | -2.00 | 0.049 |
| Comt | ENSMUSG00000000326 | -1.92 | 0.037 |
| Mlf2 | ENSMUSG00000030120 | -1.90 | 0.031 |
| Fkbp4 | ENSMUSG00000030357 | -1.76 | 0.049 |
| Ppp3r1 | ENSMUSG00000033953 | -1.57 | 0.049 |
